# A comparison of four liquid chromatography–mass spectrometry platforms for the analysis of zeranols in urine

**DOI:** 10.1007/s00216-023-04791-8

**Published:** 2023-07-11

**Authors:** Abigail Lazofsky, Anita Brinker, Zorimar Rivera-Núñez, Brian Buckley

**Affiliations:** 1 Environmental and Occupational Health Sciences Institute, Rutgers University, Piscataway, NJ 08854 USA; 2 Department of Biostatistics and Epidemiology, Rutgers School of Public Health, Rutgers University, Piscataway, NJ 08854 USA

**Keywords:** Zearalenone, Orbitrap, Synapt G1, Linear ion trap MS, Low-resolution MS, HRMS

## Abstract

Targeted biomonitoring studies quantifying the concentration of zeranols in biological matrices have focused on liquid chromatography interfaced to mass spectrometry (LC–MS). The MS platform for measurement, quadrupole, time-of-flight (ToF), ion trap, etc., is often chosen based on either sensitivity or selectivity. An instrument performance comparison of the benefits and limitations using matrix-matched standards containing 6 zeranols on 4 MS instruments, 2 low-resolution (linear ion traps), and 2 high-resolution (Orbitrap and ToF) was undertaken to identify the best measurement platform for multiple biomonitoring projects characterizing the endocrine disruptive properties of zeranols. Analytical figures of merit were calculated for each analyte to compare instrument performance across platforms. The calibration curves had correlation coefficients *r* = 0.989 ± 0.012 for all analytes and LODs and LOQs were ranked for sensitivity: Orbitrap > LTQ > LTQXL > G1 (V mode) > G1 (W mode). The Orbitrap had the smallest measured variation (lowest %CV), while the G1 had the highest. Instrumental selectivity was calculated using full width at half maximum (FWHM) and as expected, the low-resolution instruments had the broadest spectrometric peaks, concealing coeluting peaks under the same mass window as the analyte. Multiple peaks from concomitant ions, unresolved at low resolution (within a unit mass window), were present but did not match the exact mass predicted for the analyte. For example, the high-resolution platforms were able to differentiate between a concomitant peak at 319.1915 from the analyte at 319.1551, included in low-resolution quantitative analyses demonstrating the need to consider coeluting interfering ions in biomonitoring studies. Finally, a validated method using the Orbitrap was applied to human urine samples from a pilot cohort study.

**Supplementary Information:**

The online version contains supplementary material available at 10.1007/s00216-023-04791-8.

## Introduction

Zearalenone (Zen) is a naturally produced mycotoxin present in grains and in animal products [1]. A synthetic derivative and metabolite of Zen, alpha-zearalanol (Zer), is a Food and Drug Administration (FDA)–approved agent commonly used as a non-steroidal anabolic growth promoter in livestock in the USA; however, it has been banned in many countries [2]. Through activities such as agricultural migration and direct washout from manure, these compounds and their metabolites (collectively referred to as “zeranols”; Supplemental Figure S1) can contaminate environmental matrices [3, 4]. The frequency and magnitude of Zen contamination is expected to increase due to changes in global temperatures as a result of climate change [5]. The anticipated increase in environmental temperature and moisture will create ideal conditions for mold to flourish within grain products, making mycotoxic contaminants like zeranols difficult to control. Human exposure can occur through direct contact with affected environmental media, or through ingestion of contaminated food. In two small clinical studies, ZEN exposure has been associated with premature thelarche and precocious puberty [6, 7]. In contrast, our group has reported associations between prepubertal exposure to ZEN and slower growth and pubertal development in girls [8, 9]. Furthermore, the World Anti-Doping Agency (WADA) has classified Zer as a prohibited substance, acknowledging its steroidal effects in humans [10].

Prior research has documented biological levels of zeranol metabolites in the ppb range, emphasizing the need for highly sensitive analytical methods for the effective quantification of trace concentrations (9, 11–13). Chromatographic separation, either by gas (GC) or liquid chromatography (LC) interfaced with mass spectrometry (MS), is common instruments for zeranol quantitation. Several studies were published using GC instrumentation; however, more intensive sample preparation steps accompanied this analytical approach [14, 13, 15, 16]. LC techniques are more common in zeranol analysis because they are designed for analyses of polar, thermally labile compounds without derivatization [17, 18].

MS is a powerful tool when used for the accurate identification/quantification of complex compound mixtures. MS instrumentation can be separated into 2 general categories: low-resolution (LR) MS, resolution of < 2000, and high-resolution (HR) MS, resolution of ≥ 10,000 [19]—anything falling between 2000 and 10,000 can be considered “mid-resolution.” LRMS platforms are commonly used due to their affordability, ease of maintenance [20], and better sensitivity compared to HRMS platforms [21, 22]. Historically, LRMS platforms have been used for quantitative analyses of multiple known compounds because they produce reliable results, but are limited by their selectivity, as concomitant analytes differing by only 0.1 Da are not easily resolvable. While HRMS platforms are generally much less sensitive, their selectivity and high mass accuracy allow for differentiation between compounds with comparable molecular weights. Currently, HRMS platforms are still underrepresented in quantitative zeranol analysis. This work compares the benefits and limitations that MS platforms have on zeranol analysis and provides insight on method transfer, sensitivity, and repeatability for researchers using different instruments. This is the first study to quantify and compare zeranols on 4 different MS instruments within the same laboratory. Based off a comparison of performance metrics, it was determined that the Orbitrap was the best-overall platform out of those tested. We then utilized this instrument to demonstrate a validated method’s applicability in biomonitoring studies through the analysis of urine samples collected from an ongoing cohort study.

## Materials and methods

### Chemicals and reagents

Reference standards of Zen (≥ 99%), Zer (≥ 98%, HPLC grade), zearalanone (Zan; ≥ 98%), β-zearalanol (bZal; ≥ 96%, HPLC grade), α-zearalenol (aZol; ≥ 98%), and β-zearalenol (bZol; ≥ 97%) were obtained from Sigma-Aldrich. The internal standards Rac-Zearalenone-d_6_ (Zen-d_6_; ≥ 96.9%) and α-Zearalenol-d_7_ (aZol-d_7_; ≥ 98%) were obtained from Toronto Research Chemicals. Sodium acetate buffer solution (pH = 4.65, 0.2 M) was purchased from Honeywell Fluka. The enzyme β-glucuronidase from *Helix pomatia* (type HP-2, > 100,000 units/mL, sulfatase activity < 7500 units/mL) was obtained from Sigma-Aldrich. HPLC-grade methyl tert-butyl ether (MTBE) and methanol (MeOH) were purchased from VWR. HPLC-grade acetonitrile (ACN) was purchased from Honeywell. Water was obtained from a Milli-Q water purification system (Millipore).

### Stock standard solutions

Stock solutions were individually prepared by dissolving 1 mg of a pure compound in ACN. One hundred microliters of 1 mg/mL stock standard solutions of Zan, Zen, Zer, bZal, aZol, and bZol was combined and diluted with ACN to make 1 mL of a 100 µg/mL zeranol standard mixture. Similarly, 100 µL of 1 mg/mL stock standard solutions of Zen-d_6_ and aZol-d_7_ was combined and diluted to make 1 mL of a 100 µg/mL internal standard (IS) mixture. Each mixture was serially diluted down to a concentration of 0.2 µg/mL.

### Matrix-matched standards and sample preparation

For the matrix-matched standards, 0.5 mL aliquots of urine ((pooled non-human urine samples; donated anonymously and composited) were pipetted into glass tubes along with 250 µL of sodium acetate buffer, 10 µL of β-glucuronidase, and 25 µL of 0.2 µg/mL IS solution. Twenty-five microliters of different concentrations of zeranol standard mixture were then added to create standards at added concentrations ranging from 0 to 20 ng/mL in urine. To deconjugate analytes, samples were incubated overnight in a shaking water bath at 37 °C and then cooled to room temperature prior to extraction. Real-world urine samples were prepared in the same manner without the inclusion of the zeranol standard mixture. Blank urine samples were prepared as standards, with the exclusion of the zeranol standard mixture. Any background concentration levels measured in the blanks were subtracted from the measured area of the standards and samples. Blank water samples spiked with zeranol standard mixture were also analyzed. A mid-range standard at 10 ng/mL, selected for a quality control (QC) sample, was prepared in a larger quantity and used in multiple injections for analysis.

**Fig. 1:**
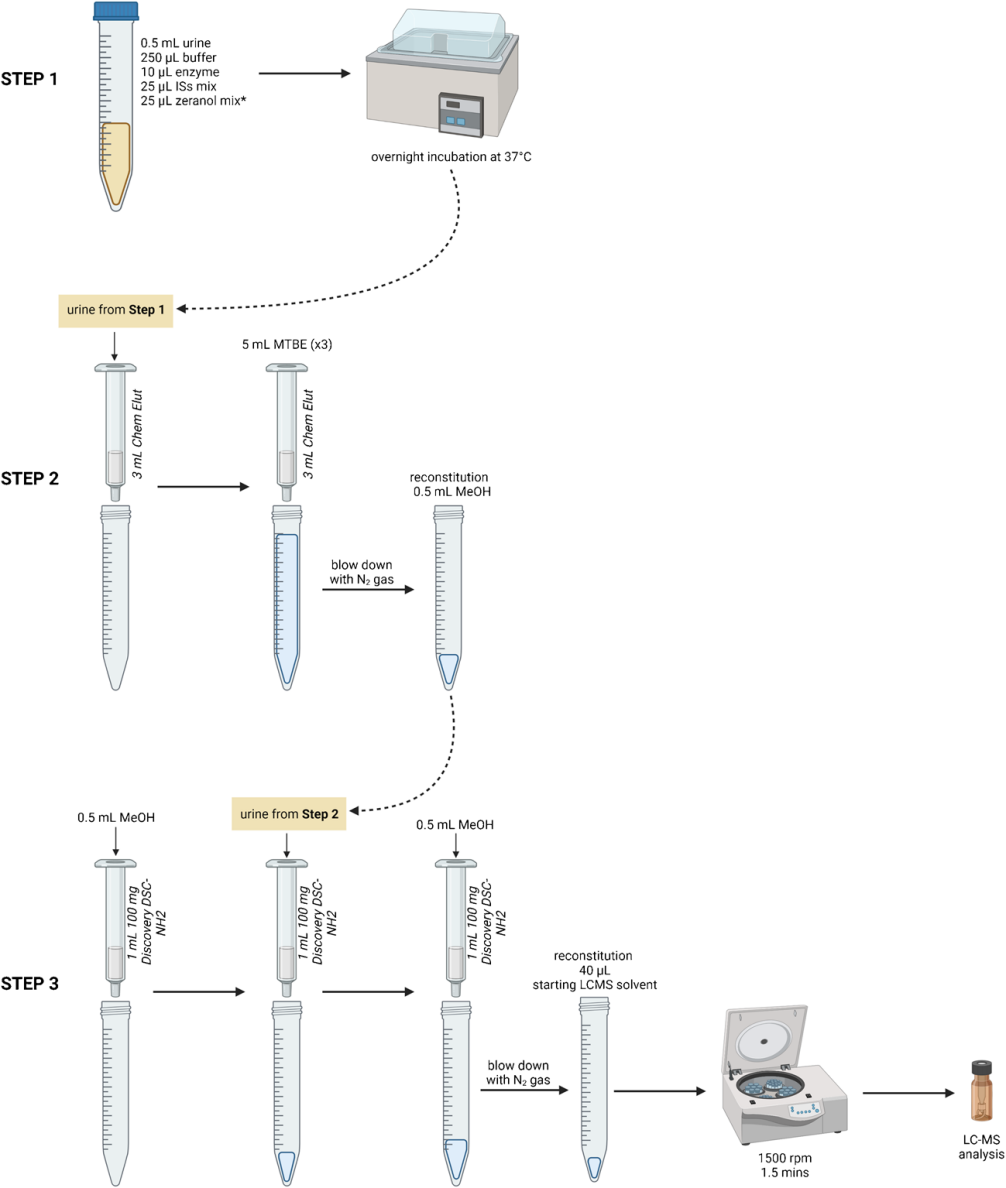
SPE protocol overview for the extraction and analysis of zeranols in urine. *Zeranol standard mixture only added to urines used for the creation of the calibration curve

All standards and samples were cleaned using solid-phase extraction (SPE). Our group has previously published earlier iterations of the sample preparation [9, 23]; an overview of the current, most optimized SPE protocol is shown in Fig. 1. Following overnight incubation, the standards and samples were loaded onto unbuffered Chem Elut cartridges (3 mL, Agilent Technologies) on top of a Visiprep™ DL SPE vacuum manifold (Sigma-Aldrich) with glass centrifuge tubes underneath. The columns were then eluted 3 times with 5 mL MTBE (15 mL total). The eluates were then dried under nitrogen, and reconstituted with 0.5 mL of MeOH. For the second extraction step, Discovery DSC-NH2 (1 mL, 100 mg, Sigma-Aldrich) cartridges were placed on the vacuum manifold. Columns were pre-conditioned with 0.5 mL of MeOH. Each sample was loaded onto a column and the analytes were eluted with 0.5 mL of MeOH and dried under nitrogen. The samples were reconstituted in 40 µL of the initial LC solvent (50:25:25 H_2_O: MeOH: ACN). Samples were then centrifuged at 1500 rpm for 1.5 min to bring down any remaining liquid from the walls of the tube. These were the conditions optimized for recoveries (see “Results: Performance Comparison: Analytical Figures of Merit”). Contents were transferred into HPLC vials for analysis.

### Instrumentation

Method optimizations were performed on 4 different LC–MS instruments. Two LR: a Thermo Scientific (TS) Surveyor HPLC interfaced to a TS LTQ Linear Ion Trap (ITMS) with an atmospheric pressure chemical ionization (APCI) source (hereafter referred to as LTQ), and a TS Accela HPLC interfaced to a TS LTQ XL Linear ITMS also with an APCI source (hereafter LTQXL). HR instrumentation included a Dionex UltiMate 3000 UPLC interfaced to a TS Q Exactive HF Hybrid Quadrupole-Orbitrap with an electrospray ionization source (ESI) (hereafter Orbi), and a Waters Acquity UPLC system interfaced to a Waters Synapt G1 High-Definition MS also with an ESI source (hereafter G1). The G1 performance was evaluated in both V and W flight path configurations. A TS BetaSil Phenyl Hexyl column (100 × 4.6 mm; 3 µm) at 35 °C with a mobile phase of H_2_O (solvent A), MeOH (solvent B), and ACN (solvent C) was used for all chromatographic separations. The gradient was as follows: 0–0.5 min 25% B and C each; 0.5–3 min linear ramp to 40% B and C each; 3–5 min linear ramp to 47.5% B and C each, 5–11 min 47.5% B and C each; 11–11.01 min linear ramp to 25% B and C each (4 min equilibration time between injections for a total run time of 15 min). A two-solvent mobile phase was created to be compatible with the binary pumping system on the G1 by combining equal fractions of solvent B:solvent C. The flow rate was 500 µL/min and the injection volume was 5 µL. Identical LC parameters were used on all 4 instruments, normalizing the chromatographic separation of the analytes and resulting in the following elution order: bZal < bZol < Zer < aZol < Zan < Zen. Mass spectral conditions were optimized for each instrument; a table containing detailed operating parameters, including operation mode, is found in Supplemental Table S1. All gas flows used nitrogen except for collision gas. All data was collected in negative ionization mode. The specific parent and product ions for both the analytes and ISs are provided in Supplemental Table S2. Data acquisition and processing were carried out with Xcalibur software for all TS platforms (v2.0.7 or v4.0), and MassLynx (v4.1) software for the Waters instrument.

### Performance comparison: analytical figures of merit

The extraction efficiency of the SPE protocol was calculated by comparing the peak areas of a post-sample preparation spiked urine sample with the area of a pre-sample preparation spiked urine sample for each of the 6 zeranol analytes at 10 ng/mL (*n* = 4). The recovery was calculated as *R*_E_ (%) = area_pre_/area_post_ × 100%. Calibration curves were calculated using peak area ratios of analytes to ISs (*y*) vs zeranol standard concentration (*x*). All standards were injected in triplicate. Linearity was evaluated over the concentration range of 0–20 ng/mL. The correlation coefficient (*r*) was measured, and the residual sum of squares (SSR) was calculated. The method sensitivity (slope) [24] was calculated using peak area ratios of analytes to ISs (*y*) vs zeranol standard concentration (*x*). Repeatability (intra-day precision) was calculated by preparing multiple matrix-matched standards at concentrations of 10 ng/mL (QC; *n* = 5–7), 2 ng/mL (*n* = 3), and 1 ng/mL (*n* = 3) and performing analysis within the same day. Reproducibility (inter-day precision) was calculated by repeating QC sample analysis on 2 different days ~ 1.5 weeks apart. Precision values of %CV < 20 were considered acceptable [25]. The limit of detection (LOD) and limit of quantitation (LOQ) were determined using standard deviation (sd_0_) of unspiked urine samples (*n* = 3) injected 5–7 times, calculated as 3sd_0_ and 10sd_0_, respectively [26]. The selectivity of the method was determined with resolution and mass accuracy for each compound. The resolution was calculated by taking the mass of the parent ion peak and dividing it by its peak width at half height (FWHM) [27], and then averaging all analytes. Mass accuracy was calculated as [(measured mass − theoretical mass)/theoretical mass)] × 10^6^ and expressed as ppm error [28]. Urinary zeranol values were corrected for urine dilution by specific gravity (SG) using the formula: mycoestrogen_SG_ _corrected_ = mycoestrogen value/[(SG − 1) × 100] [9]. The SG value of the urine used for optimization experimentation was 1.0116.

### Validation of method on the best-performing platform using real-world urine analysis of zeranols

Urine samples (*n* = 10) were collected from pregnant women (*n* = 33) enrolled in a pilot study examining prenatal exposure to zeranols in an ethnically diverse urban area of New Jersey. Samples were collected at three time points during pregnancy (18–22, 26–30, > 32 weeks). The average age for participants was 26 years, with half self-identified as Hispanic. Urine samples included here were randomly selected from all women at three time points. Samples were prepared using the aforementioned SPE protocol and analyzed using the overall best-performing MS platform, the Orbi, to corroborate its utility in the examination of real-world biological samples. Due to the anticipated low biological concentrations, validation of the method was performed including additional calibration points between 0 and 1 ng/mL using many of the same metrics as described in “Materials and methods—‘Performance comparison: analytical figures of merit.’” The average SG readings for these samples were 1.0149 ± 0.008.

## Results

### Performance comparison: analytical figures of merit

A series of identical experiments using a standard-spiked matrix was performed on each of the 4 different LC–MS platforms described above. The extraction recoveries *R*_E_ at 10 ng/mL were bZal (81.2% ± 16%; CV = 19.2%), bZol (84.0% ± 16%; CV = 19.4%), Zer (93.4% ± 12%; CV = 12.8%), aZol (91.7% ± 13%; CV = 14.3%), Zan (84.3% ± 14%; CV = 16.6%), and Zen (87.4% ± 18%; CV = 21.0%). *R*_E_ at a lower and biologically relevant concentration (0.025 ng/mL) demonstrates similar recoveries and measurement variation, indicating that the current SPE protocol is robust in its range of applicability (see Supplemental Table S3 for more details). Additionally, ISs peak area variation acted as a QC metric for method performance (CV ≤ 30%), and results further supported the use of the protocol without further optimization. We acknowledge that utilizing a mixed-sorbent SPE approach rather than a 2-step extraction may have streamlined sample preparation, but we have not explored this alternative approach.

Linearity was evaluated with the measured correlation coefficients (*r*) and the sum of squared residuals (SSR) (Table 1). Correlation coefficients of *r* = 0.989 ± 0.012 across all curves and instruments confirmed strong linear relationships (0–20 ng/mL) for each analyte. The Orbi had the best linear relationship, averaging *r* = 0.998 and SSR = 0.0268 across all zeranols. Larger SSRs for Zan on the LTQ (0.259) and G1 (both V (0.382) and W mode (4.753)) were indicative of poorer-fitting linear regression lines. A similar conclusion was observed for Zen (SSRs of 0.772 and 9.045 for G1 in V and W modes).

**Table 1:** Analytical figures of merit for each instrument (and mode) and all zeranol analytes

		LTQ	LTQXL	Orbi	G1 (V mode)	G1 (W mode)
Linearity (*r*, SSR)	*bZal*	0.998 (0.003)	0.987 (0.015)	0.999 (0.004)	0.984 (0.068)	0.991 (0.023)
	*bZol*	0.985 (0.001)	0.980 (0.001)	0.993 (0.003)	0.986 (0.002)	0.939 (0.012)
	*Zer*	0.998 (0.002)	0.993 (0.005)	0.999 (0.119)	0.987 (0.040)	0.975 (0.042)
	*aZol*	0.988 (0.005)	0.994 (0.002)	0.999 (0.006)	0.992 (0.019)	0.994 (0.024)
	*Zan*	0.996 (0.259)	0.998 (0.091)	0.999 (0.009)	0.990 (0.382)	0.991 (4.753)
	*Zen*	0.997 (0.072)	0.997 (0.081)	0.999 (0.020)	0.977 (0.772)	0.973 (9.045)
Sensitivity (peak counts/(ng/mL))	*bZal*	3430	1290	8.00 × 10^6^	7.59	0.711
	*bZol*	872	266	3.00 × 10^6^	1.58	0.189
	*Zer*	3010	1100	1.00 × 10^7^	6.49	0.593
	*aZol*	2000	707	1.00 × 10^7^	5.14	0.906
	*Zan*	2230	637	2.00 × 10^7^	12.5	2.16
	*Zen*	1440	506	3.00 × 10^7^	11.1	1.60
Repeatability 1 ng/mL (%CV)	*bZal*	13.3	9.97	5.18	11.5	10.2
	*bZol*	26.9	24.1	17.4	95.4	107
	*Zer*	22.1	5.42	3.20	0.81	13.7
	*aZol*	13.8	11.1	1.43	20.2	21.0
	*Zan*	10.2	10.6	22.20	24.9	9.28
	*Zen*	7.38	32.6	1.36	14.8	26.6
Repeatability 2 ng/mL (%CV)	*bZal*	5.01	11.0	0.63	1.38	2.16
	*bZol*	4.07	7.84	4.62	78.0	11.0
	*Zer*	8.29	2.40	0.82	12.6	10.7
	*aZol*	21.7	14.8	2.82	33.4	11.7
	*Zan*	8.84	7.42	0.33	24.7	22.0
	*Zen*	3.57	14.0	1.30	24.0	26.6
Repeatability 10 ng/mL (%CV)	*bZal*	5.39	14.7	2.98	8.60	9.95
	*bZol*	5.43	15.4	6.97	15.6	36.1
	*Zer*	6.20	8.60	2.37	10.0	10.8
	*aZol*	7.15	9.16	2.11	5.71	6.33
	*Zan*	3.69	8.50	2.06	5.15	6.24
	*Zen*	8.76	12.0	2.72	6.77	8.79
Reproducibility 10 ng/mL (%CV)	*bZal*	14.5	13.8	12.5	13.6	20.4
	*bZol*	15.3	13.7	12.6	39.1	31.3
	*Zer*	9.70	8.54	3.31	25.8	9.45
	*aZol*	12.1	8.28	3.91	24.5	7.79
	*Zan*	7.42	7.75	3.32	6.19	7.17
	*Zen*	9.65	10.0	3.22	20.4	7.03
LOD (ng/mL)	*bZal*	0.028	0.025	0.027	0.505	1.59
	*bZol*	0.242	0.990	0.120	0.793	12.1
	*Zer*	0.077	0.056	0.281	1.19	4.46
	*aZol*	0.093	0.116	0.025	0.413	1.23
	*Zan*	0.053	0.074	0.025	0.291	0.335
	*Zen*	0.202	0.154	0.022	0.551	2.62
LOQ (ng/mL)	*bZal*	0.095	0.084	0.089	1.69	5.28
	*bZol*	0.807	3.30	0.400	2.64	40.2
	*Zer*	0.257	0.187	0.936	3.98	14.9
	*aZol*	0.311	0.386	0.084	1.38	4.11
	*Zan*	0.178	0.248	0.083	0.969	1.12
	*Zen*	0.672	0.513	0.070	1.84	8.75
Resolution	*bZal*	684.5	698.2	45,880	7559	10,040
	*bZol*	625.3	602.4	48,360	6384	-
	*Zer*	720.1	642.5	47,230	8031	13,380
	*aZol*	764.5	665.1	46,930	10,640	11,400
	*Zan*	573.4	938.5	45,590	6720	10,640
	*Zen*	780.7	793.3	44,050	7049	11,330
Mass accuracy (ppm error)	*bZal*	177.1	0.623	1.25	222.0	76.3
	*bZol*	92.3	298.9	0.940	211.2	56.4
	*Zer*	177.1	248.5	0.934	201.5	76.3
	*aZol*	234.8	298.9	1.57	211.2	88.7
	*Zan*	234.8	233.7	1.25	204.9	77.7
	*Zen*	293.9	350.3	1.58	241.2	88.9

**Fig. 2:**
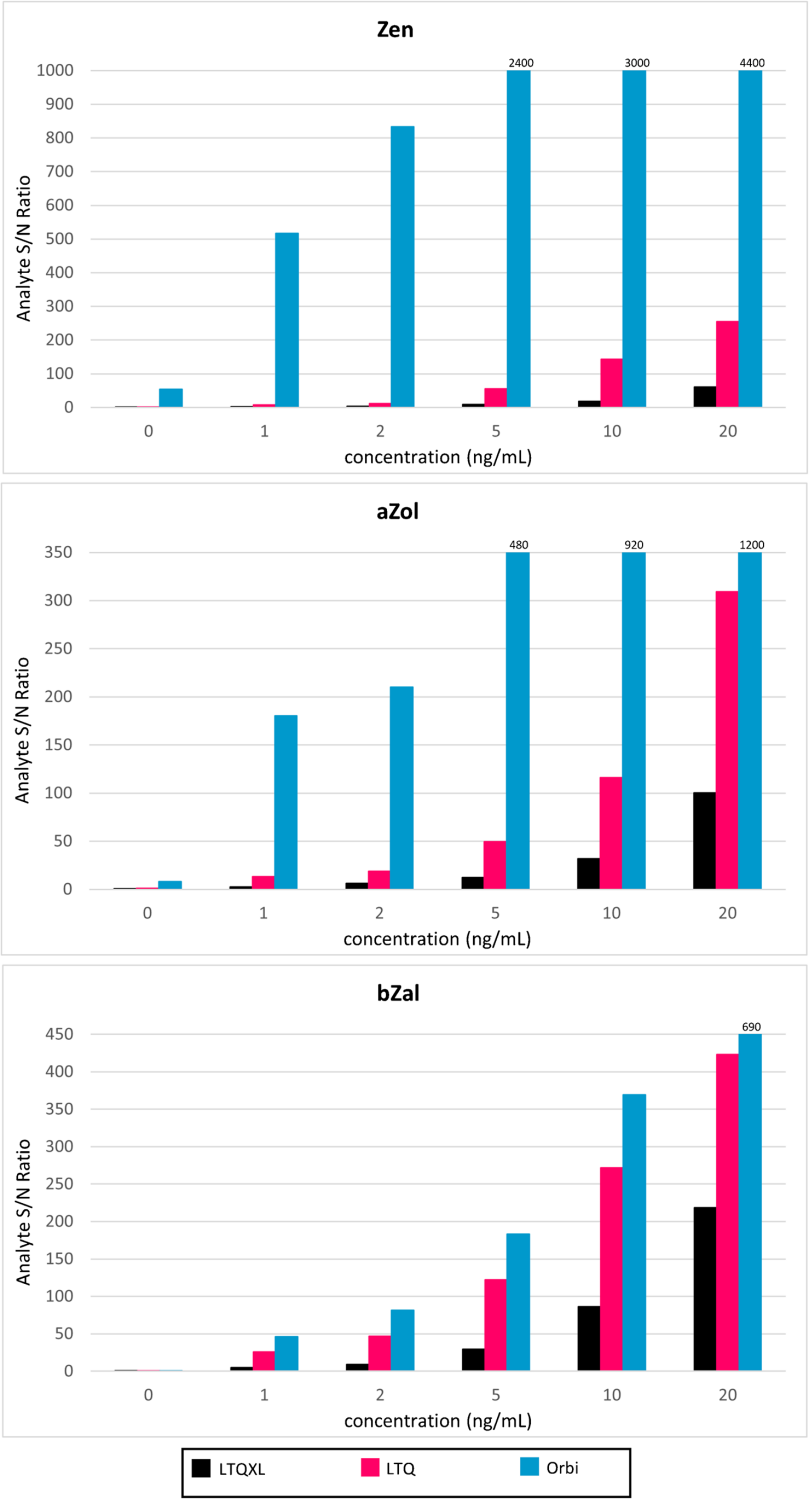
Comparison of S/N (except G1*) for all Thermo-manufactured instruments including HRMS**. For some compounds, analysis of blank matrix resulted in higher S/N values due to expected background levels present in our control group***. *G1 excluded from graphical representation due to a difference in the algorithm used for calculating S/N. **Zen (*m/z* = 317.1389), aZol (*m/z* = 319.1546), and bZal (*m/z* = 321.1702); representative compounds from each parent m/z selected arbitrarily. ***Example chromatograms of blank matrix evaluated for the above compounds can be found in Supplemental Figure S3

Sensitivities varied greatly between instruments (Table 1). The Orbi had the greatest sensitivity for all analytes, resulting in values ~ 10^6^-fold greater in comparison to the other instruments. The average sensitivity for all analytes was as follows: LTQ = 2160 ± 953, LTQXL = 751 ± 380, Orbi = 13,500,000 ± 9,790,000, G1 (V mode) = 7.40 ± 3.99, and G1 (W mode) = 1.03 ± 0.723, suggesting that across all zeranols, the G1 in W mode was the least sensitive. Higher sensitivity implies a measurable signal for small concentrations to be detected above the background or noise [29], indicating signal-to-noise ratio (S/N) is a further measure of sensitivity. Figure 2 compares the S/N of a given concentration (calculated by the Xcalibur Genesis algorithm; Supplemental Figure S2) between all Thermo-manufactured instruments; the G1 was excluded due to the difference in the manufacturer-based algorithm used to compute S/N. The Orbi S/N ranged from equivalent to 70-fold higher than the LRMS LTQ, the platform with the next-highest S/N levels, further highlighting the high sensitivity capabilities of the HRMS platform. For all platforms, most prominently the Orbi, analysis of the blank matrix resulted in elevated S/N (Supplemental Figure S3). We assume that most, if not all, individuals have had exposure to mycoestrogens resulting from lifestyle and diet habits, which was not controlled for prior to the collection of urines pooled for optimization experimentation. However, we do account for this endogenously present concentration in the creation of our standard curves.

Both repeatability and reproducibility (%CV) were also reported in Table 1. Nearly all instruments produced an “acceptable” range for both repeatability (2 ng/mL %CV = 10.3% ± 9.1%; 10 ng/mL %CV = 8.47% ± 6.4%) and reproducibility (%CV = 12.7% ± 8.5%). The G1 in V mode displayed high intra-day variability for bZol (78.0%) and aZol (33.4%) at 2 ng/mL, and in W mode for bZol at 10 ng/mL (36.1%). Both the V and W modes of the G1 produced higher inter-day variability for bZol (39.1%), Zer (25.8%), and aZol (24.5%) in V mode, and bZol (31.3%) in W mode. Additional repeatability experiments were performed at 1 ng/mL as a way to evaluate instrument performance at the lowest concentration all instruments were able to analyze. These results follow a similar trend, with %CV = 14.0% ± 8.7% and high variability occurring for bZol in both the V and W modes of the G1.

The LOD and LOQ are displayed in Table 1. The Orbi had the best LOD values for bZol (0.120 ng/mL), aZol (0.025 ng/mL), Zan (0.025 ng/mL), and Zen (0.022 ng/mL) and LOQs for bZol (0.400 ng/mL), aZol (0.084 ng/mL), Zan (0.082 ng/mL), and Zen (0.070 ng/mL). The LTQXL had the best LODs for bZal (0.025 ng/mL) and Zer (0.056 ng/mL) and LOQs for bZal (0.084 ng/mL) and Zer (0.187 ng/mL). The G1 in W mode consistently had the highest LODs for all analytes (1.234–12.06 ng/mL), although Zan had a moderately low value (0.335 ng/mL).

Individual analyte resolution and mass accuracies are reported in Table 1. The overall instrument resolution was numerically expressed by averaging the individual resolution calculations across all 6 zeranol metabolites. Instruments were reported to have the following overall resolutions: LTQ (691 ± 80.6), LTQXL (723 ± 124), Orbi (46,340 ± 1500), G1 in V mode (7731 ± 1540), and G1 in W mode (11,360 ± 1260). Supplemental Figure S4 highlights the differences in peak width across the highest-resolution platform (Orbi), a mid-resolution platform (G1 in V mode), and a LRMS (LTQXL). The Orbi had the narrowest peak width, while the LTQXL had the largest. The overall error in accuracy measurement was calculated by averaging the error calculations across the parent ions of all 6 zeranol metabolites. The mass accuracy error (ppm) were as follows: LTQ (202 ± 69.1), LTQXL (239 ± 123), Orbi (1.25 ± 0.285), G1 in V mode (215 ± 14.5), and G1 in W mode (81.6 ± 6.62).

### Validation of method on the Orbitrap MS using real-world urine analysis of zeranols

The analytical figures of merit were used to select the Orbi for a biomonitoring study having the best sensitivity, selectivity, and mass accuracy necessary for the analysis of zeranols in real-world urine samples. Once selected, validation of the method was performed across multiple days to include the range of concentrations reflective of expected real-world measurements; these results, which have been analyzed in regard to linearity, LOD, LOQ, and precision, can be found in Supplemental Table S4. To examine the effectiveness of quantifying low concentrations of zeranols, samples collected from 10 pregnant women enrolled in a cohort study examining prenatal exposure to mycoestrogens were analyzed. Table 2 displays concentrations of each zeranol metabolite as well as total mycoestrogen levels. Values that were found to be below the calculated LOD (those listed in Supplemental Table S4) have been reported as their measured values (rather than < LOD), as it is common practice for our biomonitoring group to report the measured levels (albeit, with higher uncertainty) to limit the use of imputed data that could unintentionally skew further epidemiological and statistical analyses. As the variation of these measurements is expected to have a greater contribution to the overall uncertainty, %CV has been reported alongside those values which have concentrations below the LOD; variation was calculated from a triplicate injection of each sample. All 10 samples had measurable concentrations above the LOD, for at least 2 metabolites. Zen, aZol, and bZol were the most frequently detected compounds, bZal was not detected in any sample, and Zer was identified in 1 sample. Total mycoestrogen levels ranged from 0.167 to 4.05 ng/mL.

**Table 2:** Zeranol metabolite concentrations measured using a validated method on the Thermo Q Exactive HF Hybrid Quadrupole-Orbi MS collected from a cohort of pregnant NJ women. Zeranol concentrations have been corrected for urinary dilution by specific gravity as described by Bandera et al. [9]*. For measurements below the reported LOD, %CV has been recorded as an expression of uncertainty

	*Zeranol concentration in urine (ng/mL)*						
Sample ID	bZal	bZol	Zer	aZol	Zan	Zen	∑_mycoestrogen_
1	nd	0.0454	0.0062	0.0968	nd	0.156	0.304
2	nd	0.0221	nd	0.149	nd	0.0871	0.258
3	nd	0.123	nd	0.511	nd	0.559	1.19
4	nd	0.0268	nd	0.0574	nd	**0.0832 (28%)**	0.167
5	nd	0.0247	nd	0.166	nd	0.179	0.369
6	nd	0.232	nd	0.948	**0.0215 (7.5%)**	2.85	4.05
7	nd	**0.0136 (48%)**	nd	0.122	**0.0051 (26%)**	0.185	0.326
8	nd	0.0379	nd	0.0785	**0.0020 (9.3%)**	0.139	0.258
9	nd	0.0582	nd	**0.0417 (1.6%)**	**0.0038 (6.6%)**	**0.0760 (9.9%)**	0.180
10	nd	0.0222	nd	0.0588	**0.0017 (33%)**	0.112	0.194

Bolded values (%) = values approximated below the reported LOD using integrated peak area (%CV)

*nd* non-detect; no analyte signal was detected at the retention time

∑_mycoestrogen_ = sum of all zeranol metabolites

^*^Mycoestrogen_SG corrected_ = mycoestrogen value/[(SG − 1) × 100]

## Discussion

### LRMS performance: LTQ vs LTQXL

The analyses from both LR linear ITMSs, LTQ and LTQXL, had comparable figures of merit. Average zeranol LODs on the LTQ (0.116 ng/mL) were slightly more sensitive than the calculated average on the LTQXL (0.236 ng/mL). Previous literature is limited in the use of ITMS concerning zeranol analysis. Frizzell et al. [30] used an LTQ MS to confirm the deconjugation of Zen in their reporter gene assays. They reported a general LOD of 1 ng/mL, less sensitive than LODs from this study; however, the matrix can often impact the sensitivity. The interface from each instrument contains the same general elements with a few notable differences. The LTQXL has an API tube lens and skimmer, and the LTQ has an S-lens and an exit lens [31]. The mass analyzers also have structural differences. The LTQXL contains a single 2D linear ion trap and relies on 3 DC axial trapping voltages to trap ions until they are sent to the detector. All major operation steps (ion storage, isolation, fragmentation, and scan-out) are performed in one trap [31]. The LTQ contains a dual-cell 2D linear ion trap and relies on 6 DC axial trapping voltages to create potential wells for holding ions. The ion scan-out/detection is performed in the high-pressure trap, while the initial trapping, isolation, and dissociation of the ions occur in the low-pressure trap [31]. All upgrades in electronics were introduced to increase analytical sensitivity between the variations of ITMS instrumentation, but our results show no crucial differences between the overall analytical capabilities of the LTQ and LTQXL in zeranols analysis.

### HRMS performance: Orbi vs G1

The Orbi and the G1 had markedly different analytical figures of merit calculations. The Orbi analysis resulted in better average repeatability (3.2%), reproducibility (6.5%), LOD (0.088 ng/mL), and LOQ (0.28 ng/mL) for zeranols compared to the G1 in both V and W modes. A previous report analyzing synthetic hormones in animal urine using an Orbi in targeted single ion monitoring data-dependent MS/MS acquisition mode calculated slightly elevated LODs for bZal (0.49 µg/L), Zen (0.38 µg/L), and Zer (0.35 µg/L) compared to this study [32]. Other studies relying on Orbi analysis for zeranol metabolites in various animal plasmas had calculated sensitive zeranol LODs as low as 0.01 ng/mL in chickens and 1.2 ng/mL in pigs [33, 34]. Carballo et al. [35] analyzed human urine samples for various dietary mycotoxins using an Agilent Ultra-High-Definition Accurate Mass quadrupole Time-of-Flight (ToF) MS and calculated LODs of 0.33 ng/mL and LOQs of 1 ng/mL for both Zen and aZol. While the specific ToF platform was different from the instrument used in this study, it is worth noting that those LODs were more sensitive than the values calculated using the G1 in both V and W modes.

Fourier transform ion cyclotron resonance mass analyzers, the family which the Orbi belongs to [36], are reported to have the highest resolution of all MS platforms and have very stable mass calibration, which translates to high reproducibility and mass accuracy; however, they can be limited in their abilities to exclude artifact peaks in spectra, which can lead to an accidental inclusion of a background peak for analyte peak integration [37]. Historically, ToF instruments have narrow dynamic ranges and, therefore, are limited in their use for quantitative analysis; however, they include the fastest analyzer for MS platforms, making them excellent instruments for screening experiments and mass confirmation studies [37, 38]. In the literature, comparisons of Orbi to ToF platforms have been performed in the analysis of pesticides in complex matrices like animal feeds and food products [39, 40], and pharmaceuticals from human and animal applications [41, 42], concluding that the Orbi had more favorable results. Gómez-Pérez et al. [39] analyzed over 300 veterinary drugs and pesticides, including various estrogens, on both an Orbi and a Waters Xevo Q-ToF–MS. They found that the Orbi was more sensitive, calculating better LOQs (12.5 ug/kg) compared to ToF (5–100 ug/kg) [46]. Additionally, they found that both instruments had comparable linearity, and the mass error was higher on the ToF than with the Orbi, similar to what we observed. Kaufmann and Walker 2017 also determined that the Orbi produced a higher mass accuracy in comparison to their Waters Vion Ion Mobility Q-ToF [42]. We could locate no publications comparing the performance of a G1 to other MS instruments, making this the first study explicitly reporting on its sensitivity and selectivity capabilities relative to other commonly used MS platforms.

### G1 performance: V mode vs W mode

The longer the flight path in ToF instruments, the greater the resolving power, increasing instrument selectivity and minimizing the peak width of the analyte ion and improving the mass resolution [43]. The different modes of the G1 delineate the drift paths that ions follow. The W mode is a method of enhancing spectral resolution without increasing the instrument footprint; by activating an additional ion mirror in the TOF analyzer, the flight path of the ions is effectively doubled therefore increasing the resolution capabilities [44]. The additional mirror, which almost doubles the flight path the ions must travel down before reaching the detector, is the major difference between the V and W modes on the G1 MS. The instrument optimization parameters (which are different from the method optimization parameters reported in Supplemental Table S1) are also different from each other and were determined when the instrument was first made operational. These parameters remain unchanged, regardless of the method. It is worth noting that the V and W flight paths are not universal across all ToF instruments. The G1 can use either flight path within the same platform. The next-generation instrument (Waters Synapt G2 series) only utilizes the higher resolution W flight path option. Other ToF instruments, like the Agilent 6500 Series Q-ToF LC/MS, have a V flight path. However, not all ToF use V or W configurations; ToF instruments have been built with Spiral Ion Trajectory, where an ion travels in a repeated “figure-eight” down a drift tube, resulting in a resolution of nearly 35,000 (at FWHM) [43].

Supplemental Figure S5 compares the resolution between the V mode and the W mode of the G1, highlighting the differences in peak width of the parent mass of aZol. Even though the W mode had a calculated resolution approximately 1.5 × greater than that of the V mode, there is minimal difference visually between peak widths. It is worth noting the V mode resolution calculation did not meet our previously defined qualification to be considered a HRMS platform (≥ 10,000 [19]), while the W mode sits on the lower end of high resolution. A previous study comparing two different MS platforms in a multi-residue analysis described ToF MS as a “medium–high resolution mass spectrometer” [39], suggesting that our resolution calculations, while lower than the ideal values reported by the manufacturer [45], still fall within an expected resolution range for the platform type.

The high variability and increased mass accuracy error of the G1 measurements in both modes is most likely a result of ion drift, a continuous error that commonly occurs in ToF instruments. Flight path length can change as the tube undergoes subtle thermal expansion and contraction related to ambient temperature changes in the lab [46]. Slight dimensional changes in the flight path can heavily impact the observed ions, which in turn can influence quantitative analysis. Utilization of a lock mass, a known peak in a spectrum, would allow for *m/z* adjustments to be made to correct for minor drift in instrument stability. The G1 is equipped with a LockSpray, which allows for simultaneous injection of a reference lock mass (i.e., leucine enkephalin) to continuously correct for drift over the course of the analytical run [47]. While generally recommended, we did not find the expected improvements with our studies, so it was not employed for our studies. We believe that this was a unique occurrence specific to our instrument, and do not expect this to be the case for all instruments that use a lock mass.

### Performance of LRMS vs HRMS

Overall, the G1 platform in both V and W modes generally had the highest measurement variation and the least sensitive LODs and LOQs. The other HRMS, the Orbi, was consistently the top performer, with the lowest measurement variation and average LODs and LOQs. Romero-González et al. [47] compared the performance of an LRMS platform (Waters TQD tandem quadrupole MS), a ToF (Waters QqToF), and an Orbi instrument for the qualitative screening of food contaminants in milk and found that the Orbi had the smallest uncertainty ranges, resulting in a decrease in false positive and false negative identifications. Additionally, their ToF produced better LOQs than their LRMS analyzer. These findings are attributed to the increased resolving power of HRMS platforms. While not quantitative, they similarly determined that the Orbi was the most appropriate for real-world samples. Interestingly, both the LTQ and the LTQXL had comparable sensitivity to the Orbi, with similar LODs for bZal and better for Zer.

**Fig. 3:**
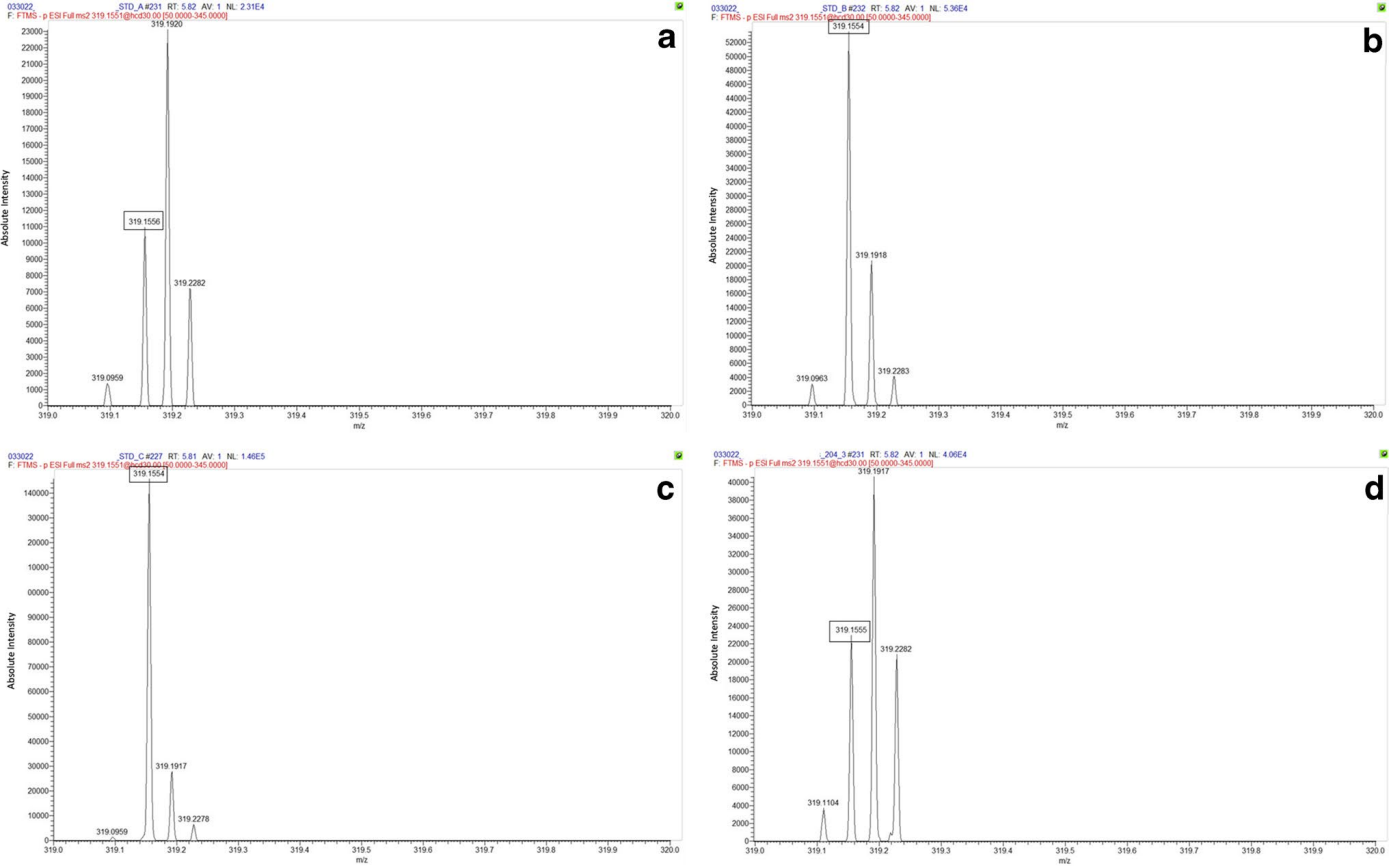
bZol parent ion peak as measured on the Orbi at **a** 0.05 ng/mL, **b** 0.5 ng/mL, **c** 1 ng/mL standard concentrations, and **d** pregnancy cohort sample calculated to be 0.1323 ng/mL. The Orbi clearly resolved 2 peaks within the expected zeranol metabolite unit mass window, corresponding with the analyte parent ion (319.1551-boxed value) and a coeluting unknown background peak (319.1915). This unknown peak is not observed in the spectrum of zeranol standard in solvent, confirming that it is related to the urine matrix. Because these peaks are resolved, only the accurate *m/z* is included in quantitative analysis

**Fig. 4:**
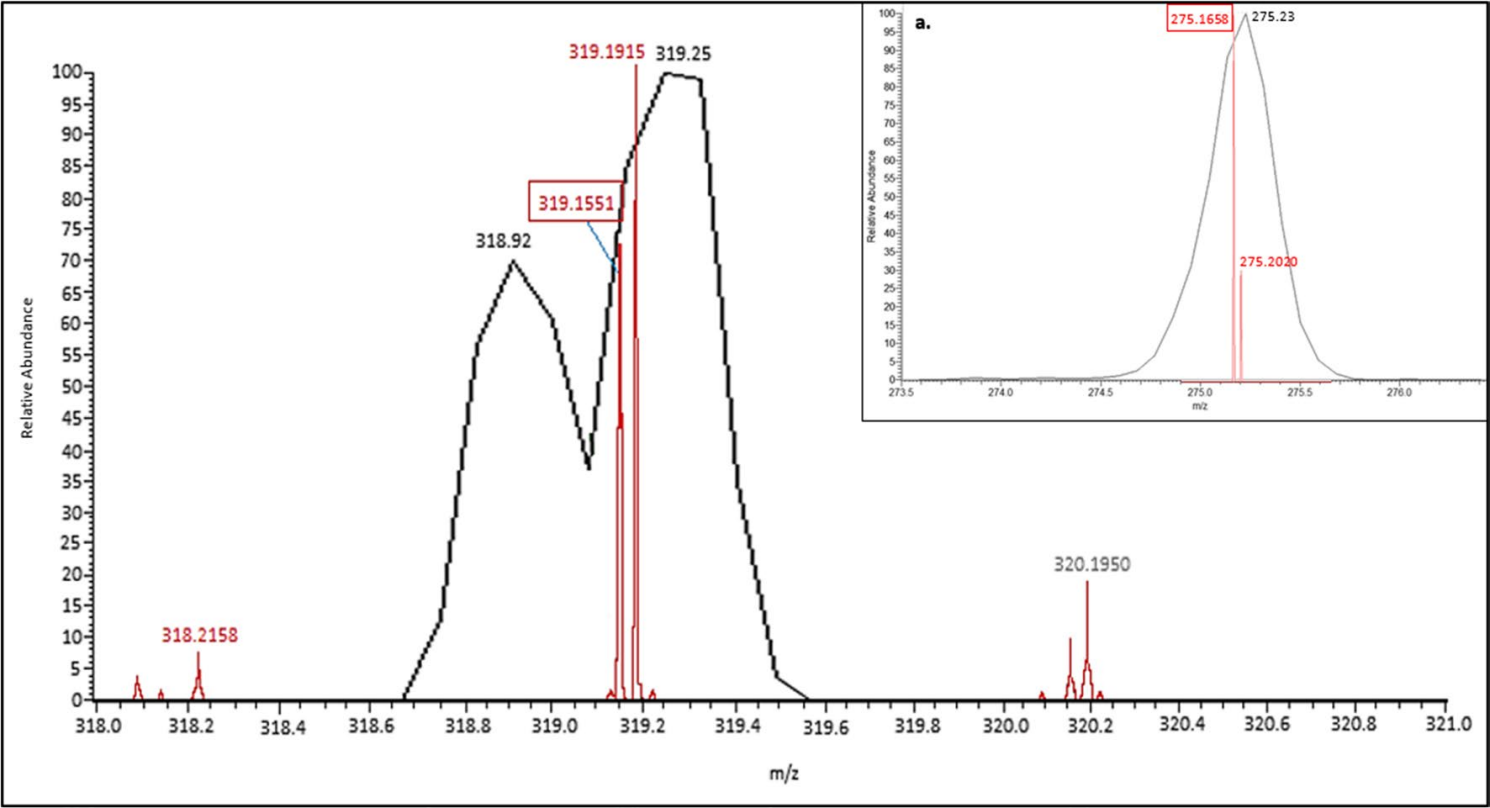
The Orbi identified 2 distinct peaks within a single Da mass window, corresponding to the aZol parent ion (319.1551-boxed value) and an unknown contaminant (319.1915). This trend can also be observed with the **a** major corresponding fragmentation peak at 275.1658 vs the contaminant fragment at 275.2020. The LR LTQ *m/z* at 319.25 is too broad to resolve these 2 peaks from one another; demonstrating possible background integration bias introduced using LRMS

Figure 3 displays the parent mass of bZol, measured on the Orbi. Two distinct *m/z* ions were present within a single mass window of 319: 319.1551, the mass of aZol, and 319.1915, a contaminant of unknown identity. This pattern of multiple peaks within a single mass unit was also seen in the spectra of Zen, aZol, bZal, and the 2 ISs; similar interfering mass ions were also observed in the fragment spectra. We further established that the relationship of the unknown peak was not a result of the zeranol standard itself through the analysis of a blank water sample spiked with zeranol mixture. It was confirmed that only the expected analyte peak at *m/z* 319.1551 was detected within the water sample. The Orbi’s resolving power allowed for discrimination, ensuring that corresponding peak areas are properly measured and analytes quantified without interference within the urine samples. We confirmed that the concomitant ion was not included in the peak integrations by narrowing mass window ranges in the quantitation method, allowing only the analyte of interest *m/z* to be included. LR platforms do not allow a narrow enough window to separate these coeluting *m/z* peaks. This is key when analyzing real-world urine samples; the broader mass window of the LTQ (Fig. 4) integrates both the analyte and the interfering background ion peaks for both the parent and product mass window for aZol. Including the background ion peak in the final peak area quantitation possibly overestimates the analyte concentration.

### Previously reported triple quadrupole performance vs HRMS

The gold standard in contamination analysis uses unit resolution tandem MS from triple quadrupole analyzers because they produce analytical sensitivity and selectivity that meet regulatory requirements [48]. Martins et al. [49] relied on a Xevo TQS (Waters) for mycotoxin analysis in urine collected from a Portuguese cohort. Their LODs for 10 different zeranol metabolites included bZal (1.6 ng/mL), bZol (0.91 ng/mL), Zer (1.12 ng/mL), aZol (0.61 ng/mL), Zan (0.15 ng/mL), and Zen (0.2 ng/mL). Both the Orbi and the G1 (V mode) used in our study had better or comparable LODs. Another study conducted by Kaklamanos et al. [50] which utilized a ThermoElectron TSQ Quantum AM MS for the analysis of anabolic steroids in bovine urine had calculated detection limits of ≤ 0.28 ng/mL for the same 6 zeranols. These results were comparable to the calculated LODs determined from our LRMS platforms, and better than those for the G1. Jia et al. [51] determined an LOD of 0.1 µg/kg for Zen in bovine milk using a Qtrap 6500 + triple quadrupole ITMS. Matraszek-Zuchowska et al. [52] quantified zeranol metabolites in animal urine using a Linear Ion Trap Quadrupole QTRAP5500 MS (Sciex) produced LODs of Zer (0.04 µg/L), bZal (0.10 µg/L), aZol (0.11 µg/L), bZol (0.13 µg/L), Zan (0.11 µg/L), and Zen (0.18 µg/L). Our instruments/methods were more sensitive for all zeranol metabolites except Zer and bZol, but still fell within a similar range. Table 3 details additional publications utilizing triple quadrupole platforms; more detailed information is found in Supplemental Table S5. Matrices examined include animal-derived foods, and both livestock and human biological samples. While it is understood that matrix type will play a role in sensitivity (and selectivity), the LOD values are highly reflective of each instrument’s capability. The reported sensitivities fell within comparable ranges, but LRMS can increase the risk of concomitant ion interference from coeluting compounds within the larger mass window.

**Table 3:** Table of previously published detection limits in ng/ml for the analysis of zeranol mycoestrogens by LC-triple quadrupole MS instrumentation

Reference	[53]	[54]	[55]	[56]	[57]	[35]	[49]	[13]
Matrix analyzed	Bovine urine	Bovine follicular fluids	Animal-derived foods (i.e., pork, eggs, milk)	Animal urine	Human urine	Homogenized pork, fish, milk, liver	Human urine	Human serum
MS instrumentation	Micromass Quattro Ultima tandem MS (Waters)	API 2000 (AB Sciex)	TSQ quantum triple stage quadrupole (TS)	Linear ion trap quadrupole QTRAP5500 (Sciex)	Varian 1200-L Quadrupole (Agilent)	API4000 MS (AB Sciex)	Quattro XEVO MS (Waters)	Xevo TQS tandem quadrupole MS (Waters)
LOD (ng/mL)								
*Zan*	0.12		0.1	0.11		0.02	0.15	0.03
*Zer*	0.18		0.1	0.04		0.03	1.12	0.04
*bZal*	0.3		0.1	0.10		0.03	1.6	0.02
*Zen*	0.03	0.01	0.1	0.18	0.002	0.03	0.2	0.02
*aZol*	0.03	0.02	0.1	0.11	0.01	0.04	0.61	0.04
*bZol*	0.02	0.02	0.1	0.13	0.01	0.04	0.91	0.06

Much of the literature exploring MS performance in the quantitative analysis focused on comparing quadrupole or trap MS platforms to either Orbi or ToF MS. Vanhaecke et al. [58] compared an Orbi to a TSQ Vantage triple quadrupole MS for the quantitative analysis of 34 anabolic steroids in meat. They calculated more sensitive decision limits (which were reported in lieu of LODs) for Zer and bZal using the LR triple quadrupole than the HRMS; a majority of the other compounds analyzed followed the same trend. Another study conducted by De Baere et al. [40] analyzing zeranol metabolite concentrations in animal plasma on both an Orbi and a triple quadrupole MS found that both platforms had comparable sensitivities, with 3 zeranol metabolites (Zen, Zer, Zan) having better LODs on the LRMS, and 3 (aZol, bZol, bZal) having better LODs on the HRMS. Flasch et al. [21] reported that their LR QTrap 6500 had better LOQs than their Orbi, suggesting that HRMS was not as effective at quantifying a broad range of low-level xenobiotics. However, the Orbi used in our study was still more sensitive than the Orbi used in the Flasch study. These studies highlight the major advantage of the Orbi, its high resolution and mass accuracy, which contributed heavily to its selectivity abilities. Triple quadrupole MSs fall under the category of LRMSs and generally provide much better sensitivity than many other platforms and are frequently used for targeted quantitation experiments. Unfortunately, we were only able to compare the instruments we had in house, and while linear ion trap instruments have similar behaviors and sensitivities to triple quadrupole instruments, a comparison to a triple quadrupole instrument may have produced a different conclusion.

Studies exploring the difference between ToF analyzers and LR trap-based instruments conclude that quantitative capabilities are similar between both instrument types, while ToF has a higher capacity for compound identification [59, 60]. ToF instruments collect all ionic data which is subsequently filtered via the software, while LR trap instruments filter analytes by mass during the collection/storage process. Ion trap instruments select only certain masses to be trapped (remain in a stable trajectory in the trap) based on the method, causing those not selected to become unstable and ejected from the trap. These masses are not retained, nor can they be viewed by the detector. Ion trap instruments are subject to space-charge limits that degrade the quality of the spectrum and create peak broadening when too many ions are stored within the trap. ToF instruments do not suffer from space-charge limits during the separation and have a much larger *m/z* range because they do not store ions in a confined volume after they are introduced to the mass spectrometer, allowing for a broader analysis more conducive with suspect screening and compound identification.

### Other considerations impacting performance

It is noted that both APCI and ESI sources were used, with the former being paired with LRMS and the latter with HRMS instrumentation. When selecting an ionization source to use for each platform, we chose the source that each instrument had been optimized with within our facilities. We acknowledge that the choice of ionization source could have some impact on overall method sensitivity; however, because we observed substantial differences in method performance between instruments, we do not believe that the ionization source was the driving force. Supplemental Table S6 displays the calculated LODs for both LRMS platforms and the Orbi using the APCI source. These results indicate that the Orbi’s LODs are still more sensitive than the LRMS platforms, suggesting that its sensitivity capabilities are more-so a function of the instrument itself.

An additional experiment was performed using each ionization source on the Orbi, indicating that the calculated LODs using an ESI were generally equivalent to 10 × more sensitive than those obtained using APCI (Supplemental Table S7). However, this is not the only performance aspect that we must keep in mind while selecting an ionization source to use: variability in signal is also important. Generally, this same experiment resulted in lower peak area variability using the APCI source in comparison to the ESI source at higher concentrations of 0.25 ng/mL, while the opposite reigned true at the lower concentration of 0.025 ng/mL (Supplemental Table S8). Therefore, the ionization source selection is more dependent on which factors the researcher deems most important (i.e., sensitivity or variation), and does not necessarily require consistent use across all platforms to obtain the most optimal results for the platform of choice. For the purpose of this study, we believed sensitivity was the more important attribute. These experiments also indicated no significant differences in each source’s ability to ionize zeranols, further demonstrating that the instrument selectivity of Orbi HRMS was responsible for the differentiation of analyte peaks from coeluting background or contaminant ions.

### Validation of method on the Orbitrap MS using real-world urine analysis of zeranols

There are few publications that analyze zeranol metabolites in urine collected from pregnant women. Ali and Degen [61] analyzed 20 urine samples collected from 20 pregnant women from Bangladesh. They reported average concentrations of Zen (0.057 ng/mL), aZol (0.151 ng/mL), and bZol (0.055 ng/mL). Another study analyzing urine collected from a cohort of 30 pregnant women from Salt Lake City, Utah reported an average concentration of Zen (0.10 µg/L) and aZol (0.11 µg/L); all other metabolites were undetected [62]. Similar to these studies, Zen, aZol, and bZol were most frequently detected in our cohort. In comparison to these two studies, the average concentrations reported in the NJ cohort were slightly higher. More samples would need to be analyzed to further explore the anticipated exposure in pregnant women (currently in progress).

## Conclusion

The performance of 4 different MS platforms in the analysis of zeranol mycoestrogens in urine was compared. Identical experiments on all instruments were performed with analytical figures of merit calculations to rank instrument operation. Overall, the HRMS Orbi provided the best sensitivity, selectivity, and mass accuracy for trace analysis of zeranols in urine. The Orbi was then used for the analysis of urine samples collected from a cohort of pregnant NJ women, where all 10 had concentrations measured above the LODs. LRMS are still the most utilized instruments for quantitation, generally either because of their sensitivity or cost. They do however fall short in their ability to differentiate from coeluting compounds possibility artificially inflating their sensitivity. The HRMS platforms examined here provided sensitivity (in the Orbi) and increased resolution required for discrimination of analyte from coeluting concomitant ions found in the same LRMS mass windows, an essential consideration for anyone performing biomonitoring studies.

## Supplementary Information

Below is the link to the electronic supplementary material.

Supplementary file1 (DOCX 1.71 MB)

## Data Availability

N/a.

## Code availability

N/a.
