# Supplementary material for: A comparison of four liquid chromatography–mass spectrometry platforms for the analysis of zeranols in urine
Source: Anal Bioanal Chem. Author manuscript; Available in PMC 2023 Sep 27. (PMC10386926; doi:10.1007/s00216-023-04791-8)
Supplement: suplemment [file NIHMS1917720-supplement-suplemment.docx]

**Supplemental Information**

**A Comparison of Four Liquid Chromatography-Mass Spectrometry Platforms for the Analysis of Zeranols in Urine**

Abigail Lazofsky^a^, Anita Brinker^a^, Zorimar Rivera-Núñez^ab^, Brian Buckley^a^*

^a^ Environmental and Occupational Health Sciences Institute, Rutgers University, Piscataway, NJ, 08854, USA

^b^ Department of Biostatistics and Epidemiology, Rutgers School of Public Health, Rutgers University, Piscataway, NJ, 08854, USA

*Corresponding author; email- [bbuckley@eohsi.rutgers.edu](mailto:bbuckley@eohsi.rutgers.edu)

**Contents**

Figure S1: *Zearalenone and its metabolites* ...……………...……………………………………………………… 3

Figure S2: *Measurement of S/N using Genesis algorithm* ………………………………………………………..... 3

Figure S3: *Blank Urine S/N* …..…………………………………………………………………………………….. 4

Figure S4: *Resolution of LTQXL vs Orbi vs G1*…..………………………………………………………………… 5

Figure S5: *Resolution of V vs W mode*……………………………………………………………………………… 5

Table S1: *Optimized MS conditions*………………………………...………………………………………………. 6

Table S2: *Parent and product ions*…….……………………………………...……………………………………. 7

Table S3: *SPE extraction recoveries*……….………………………………………………………………………. 7

Table S4: *Orbi Method Validation Results for Real World Sample Analysis …………………………………………..* 8

Table S5: *Previous Literature*………………………………………………………………………………………. 9

Table S6: *LODs of LRMS vs. HRMS using APCI* …….…………………………………………………………... 14

Table S7: *LODs of ESI vs. APCI using the Orbitrap MS* …….…………………………………………………... 14

Table S8: *Peak Variation of ESI vs. APCI using the Orbitrap MS* ...……………………………………………... 14


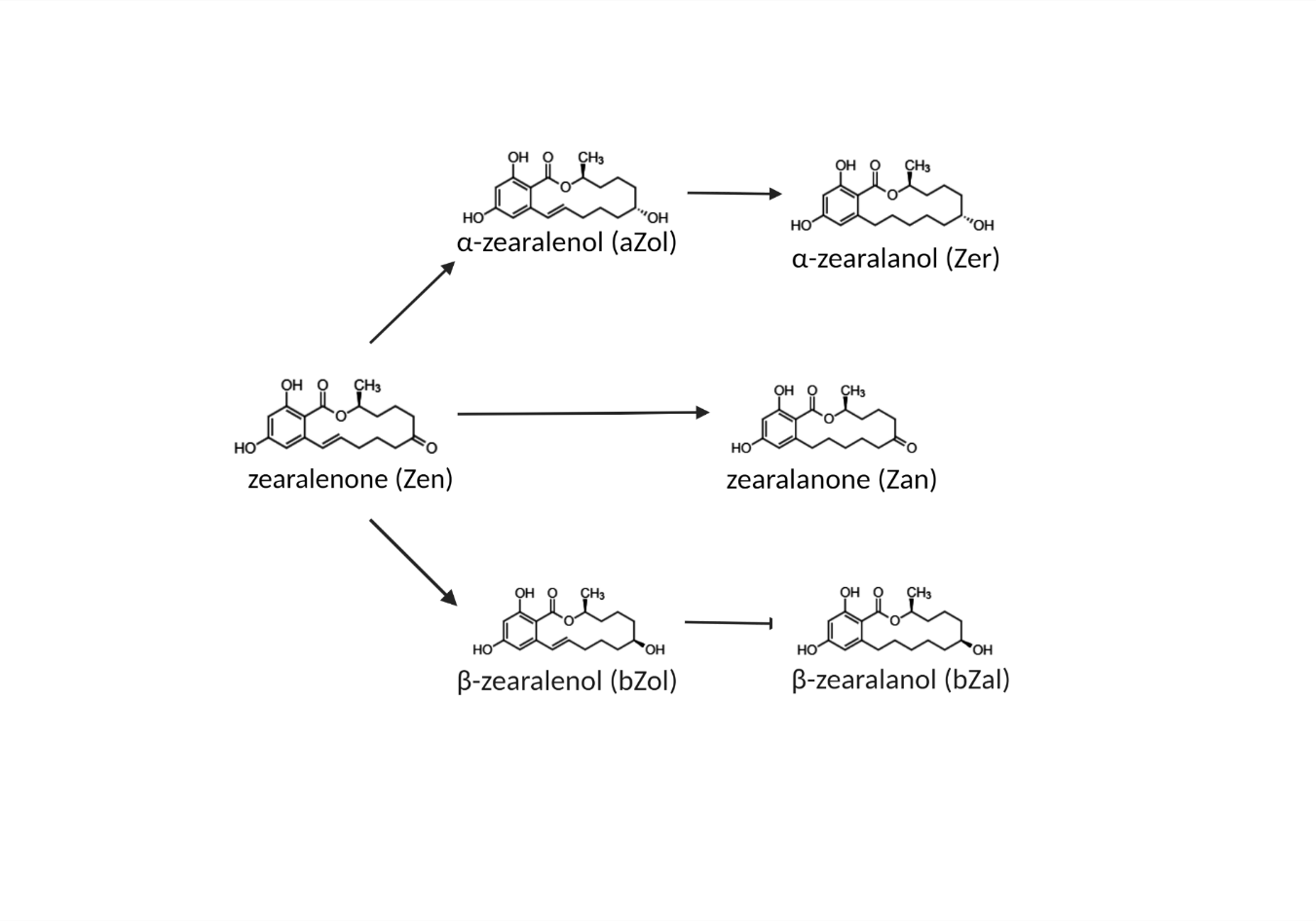
**Supplemental Figure S1.** Zearalenone (Zen) and its metabolites (collectively referred to as “zeranols” in this manuscript).


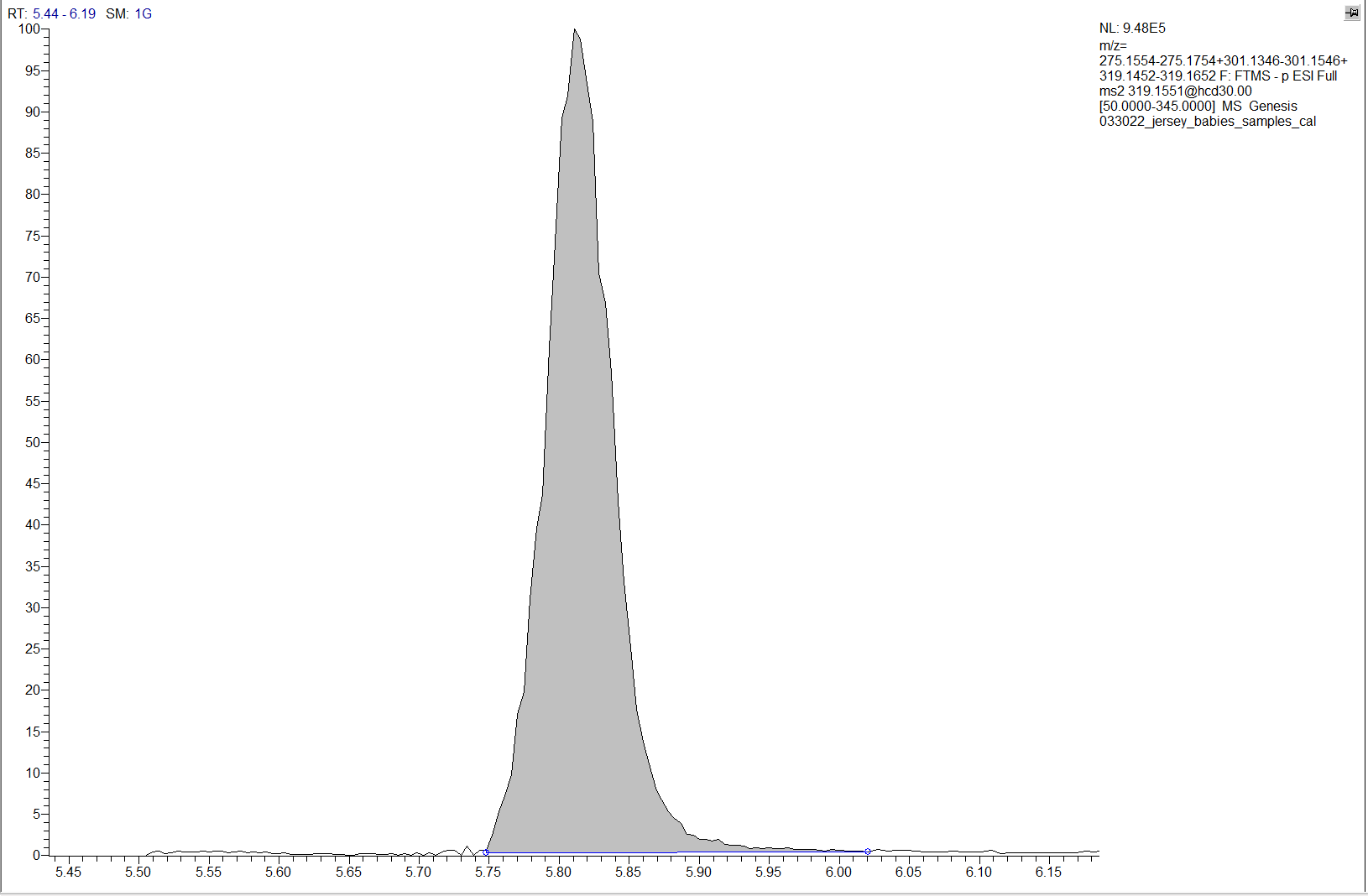

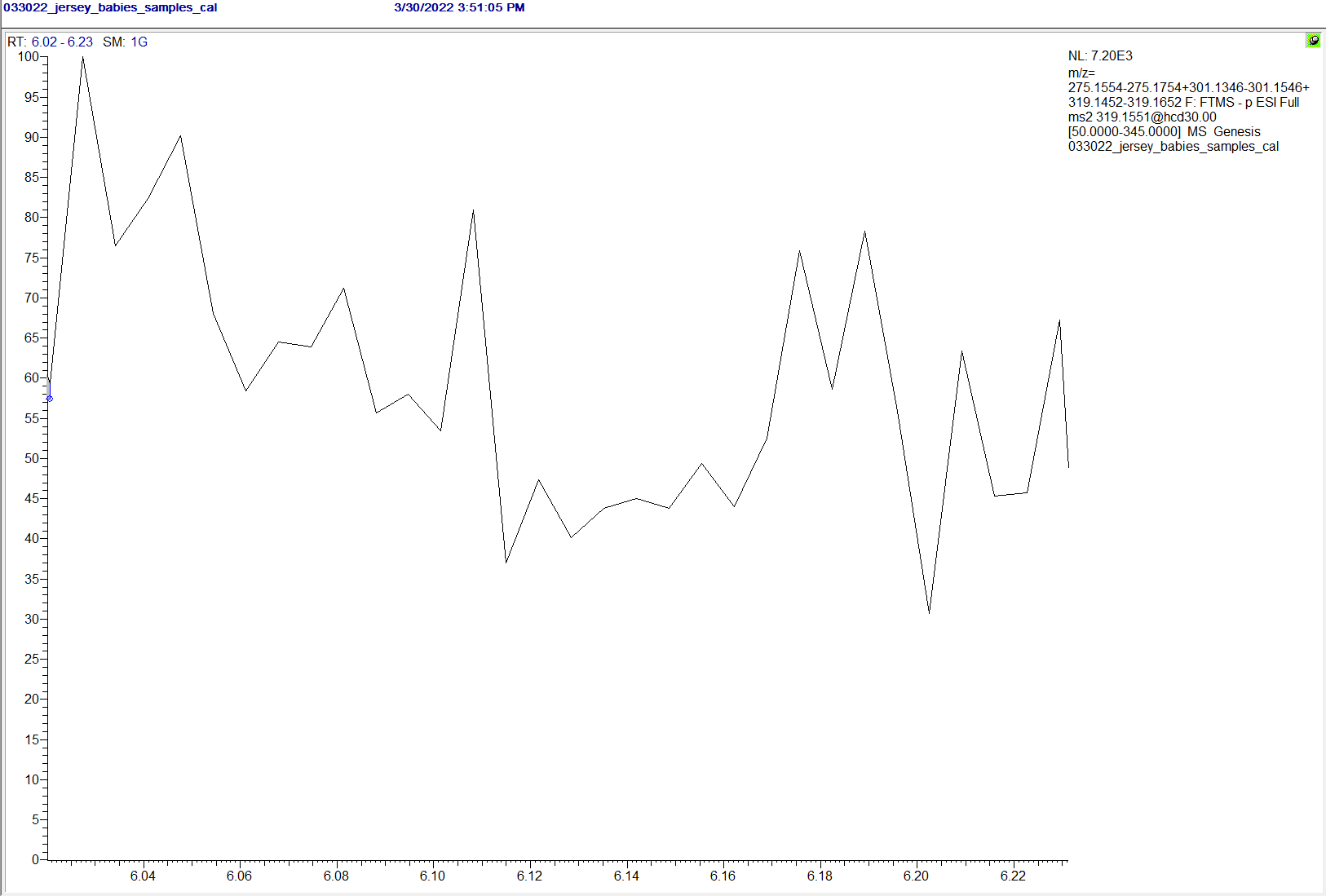


Relative abundance (%)


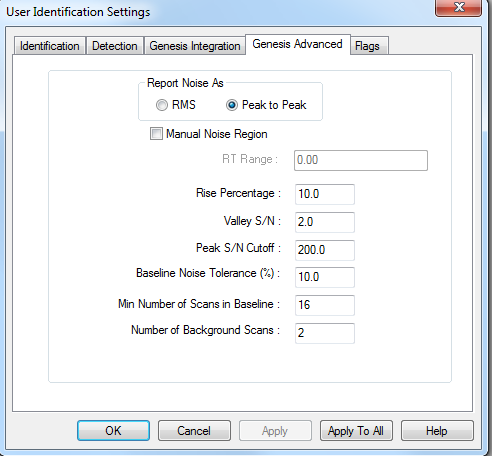


**N**

**Supplemental Figure S2.** Xcalibur Genesis algorithm used to determine S/N ratio for Thermo Fischer instrumentation. The Genesis algorithm, whose processing parameters are shown on the left, uses a “peak-to-peak” approach for calculating noise. This value is determined by taking a section of noise (time increment determined either manually or by program default) and measuring the difference between the highest spike in noise (top red dashed line) and the lowest spike in noise (bottom red dashed line) to get a reported noise level (solid red middle line; “N”).


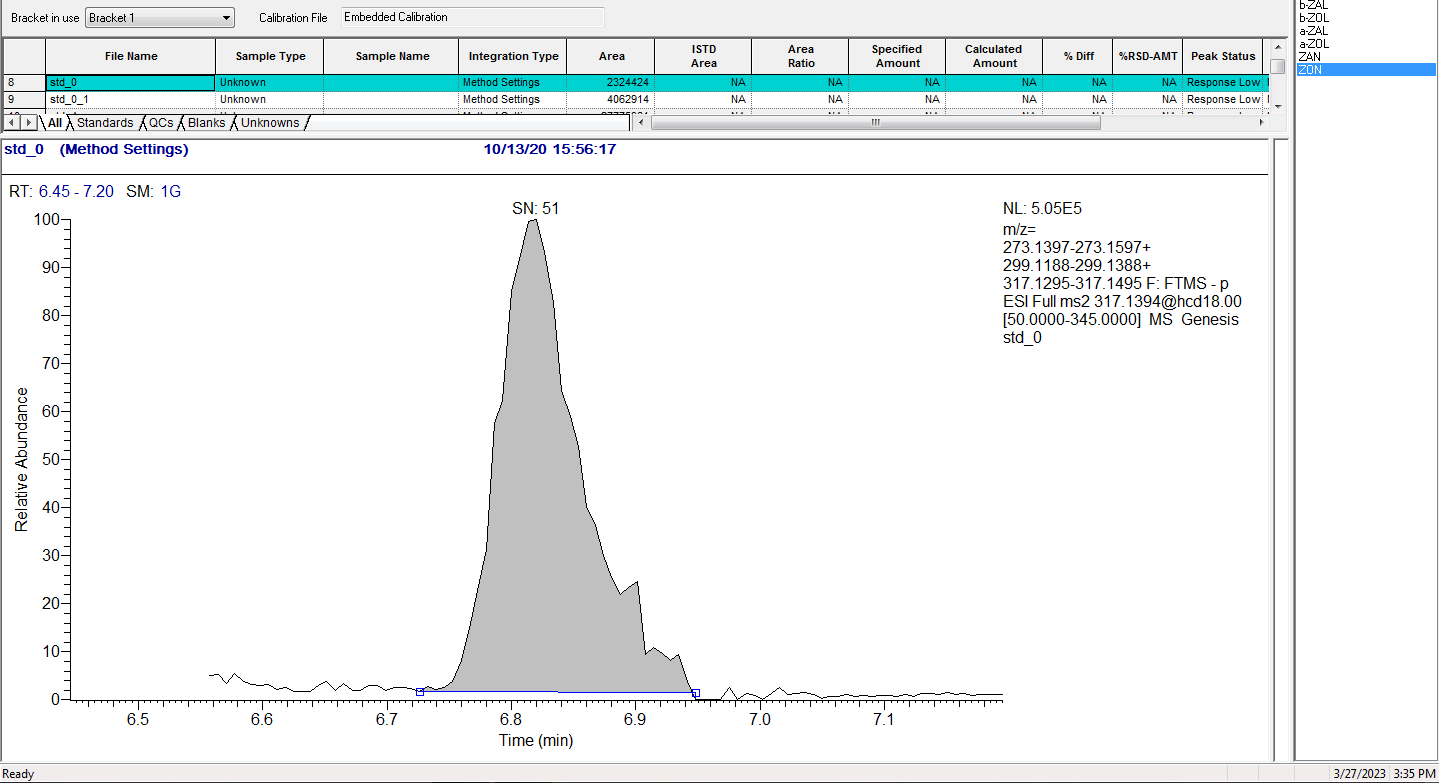

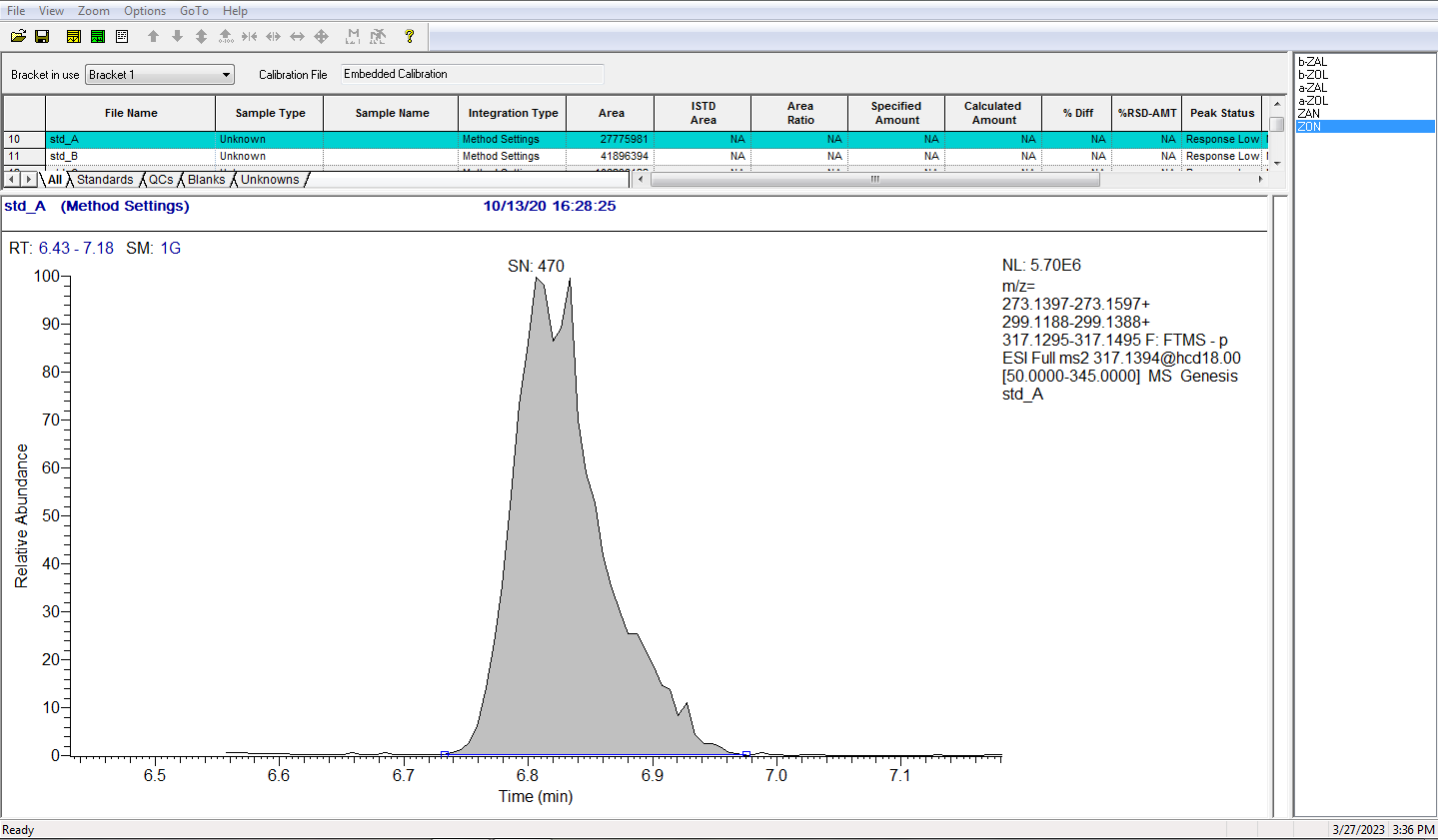


**urine blank**

**1 ng/mL spiked urine**

**Zen**


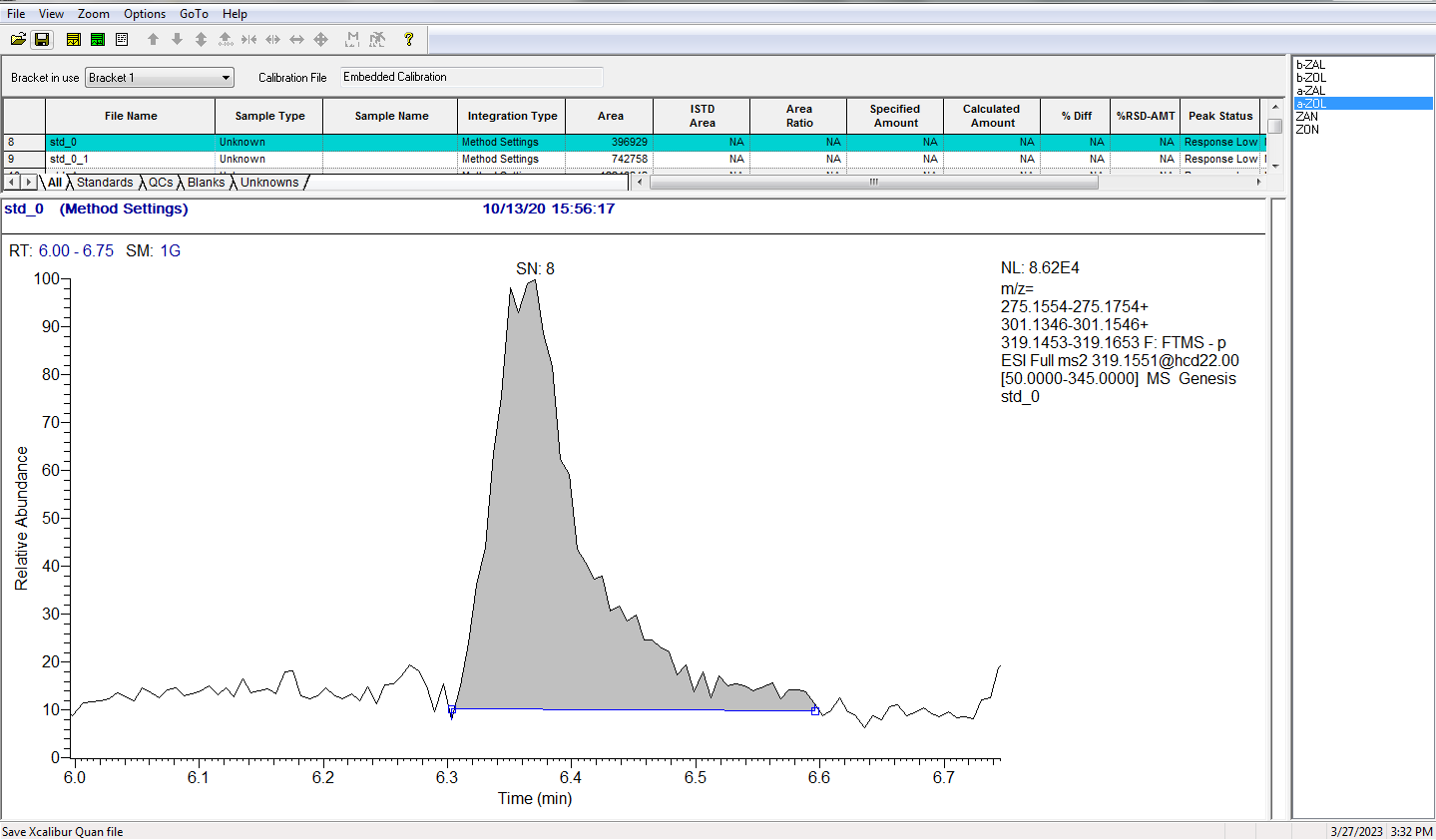

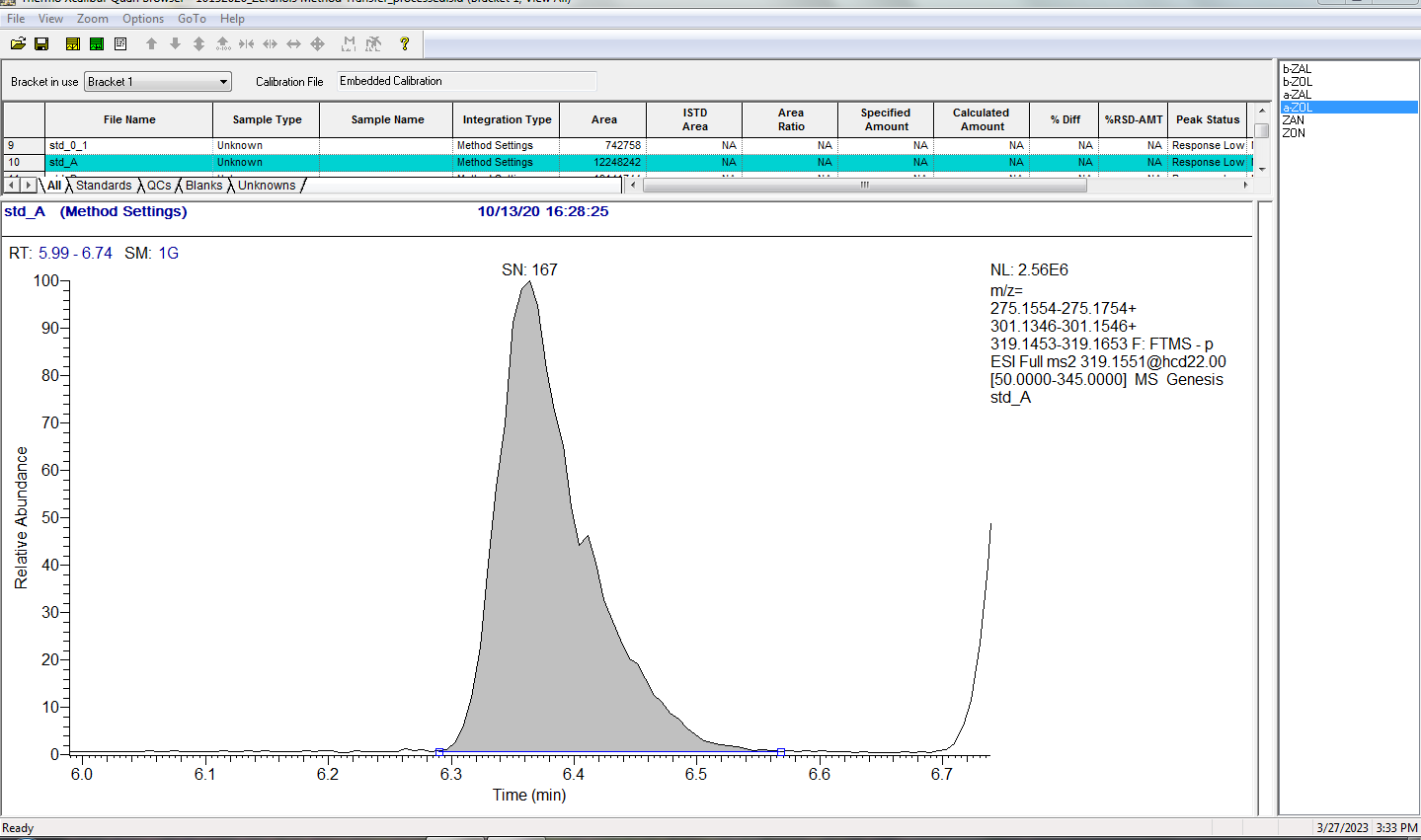


**aZol**


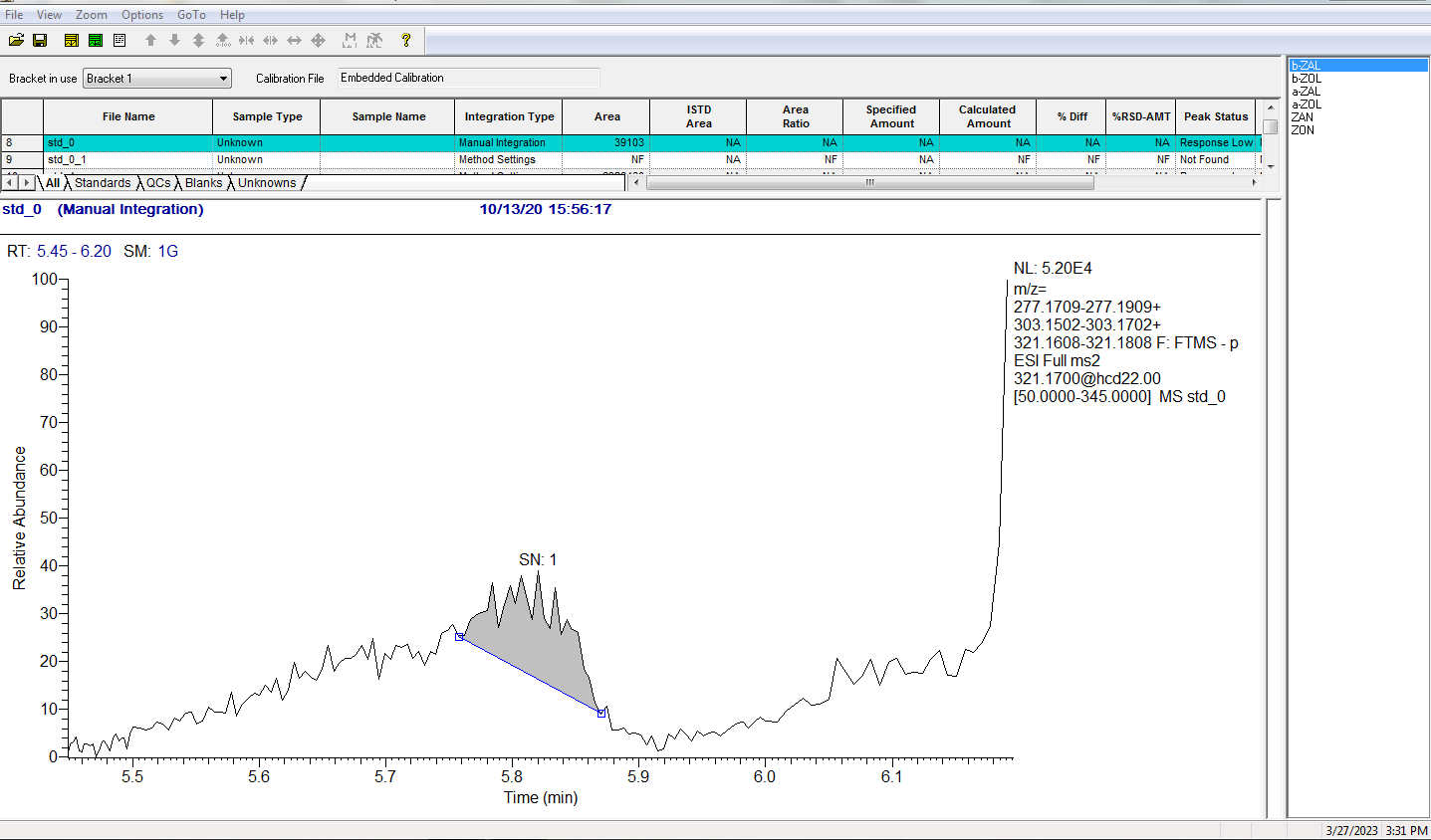

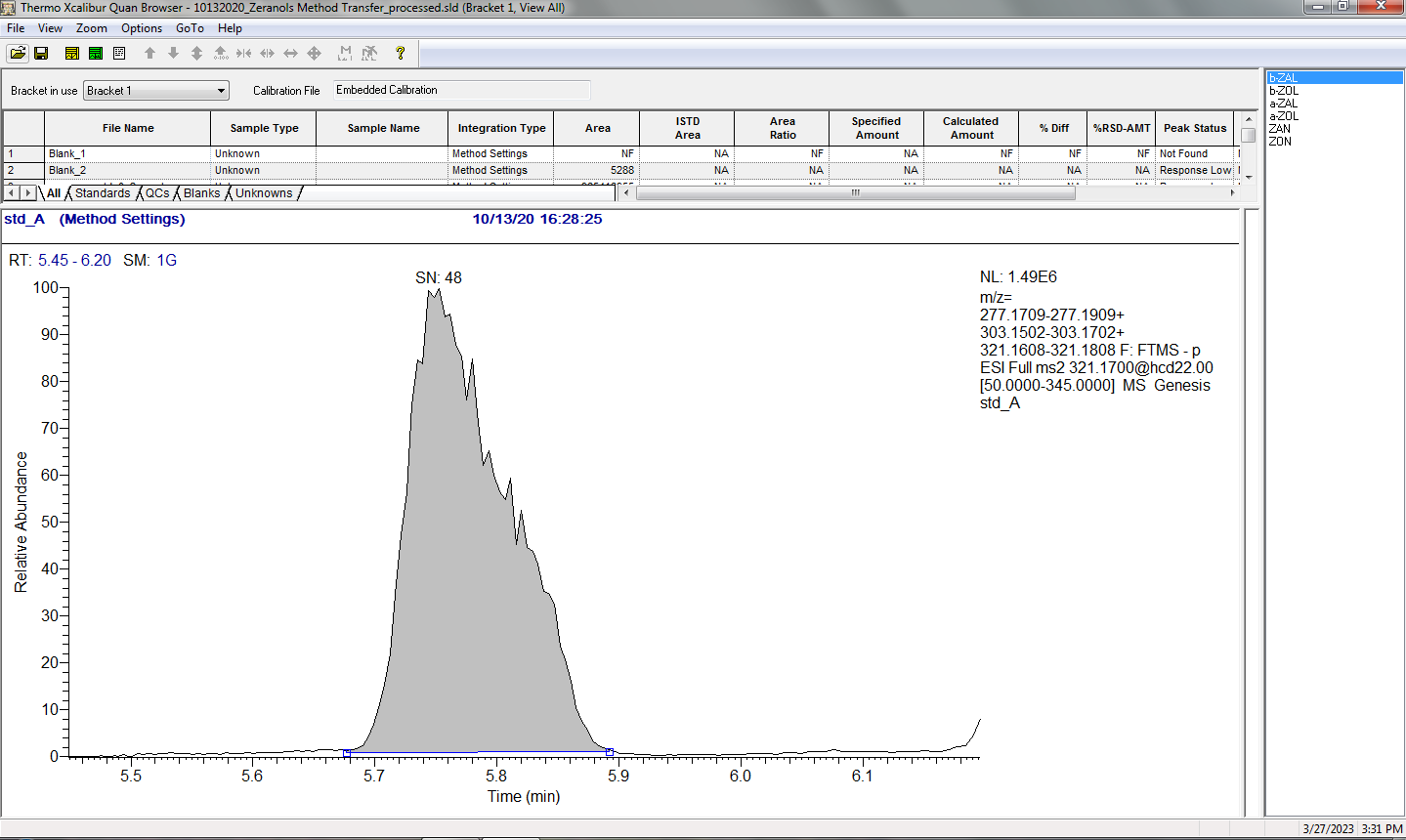


**bZal**

**Supplemental Figure S3.** Example chromatograms taken from the analyses of both blank urine and a 1 ng/mL matrix matched standard on the Orbi. Results indicate that for some compounds like Zen and aZol, high S/N readings are measured in the blank matrix. This is expected; it is assumed that most individuals have had exposure to zeranols throughout their lifetime (i.e., diet). Any background concentration was corrected for during the creation of the calibration curves.


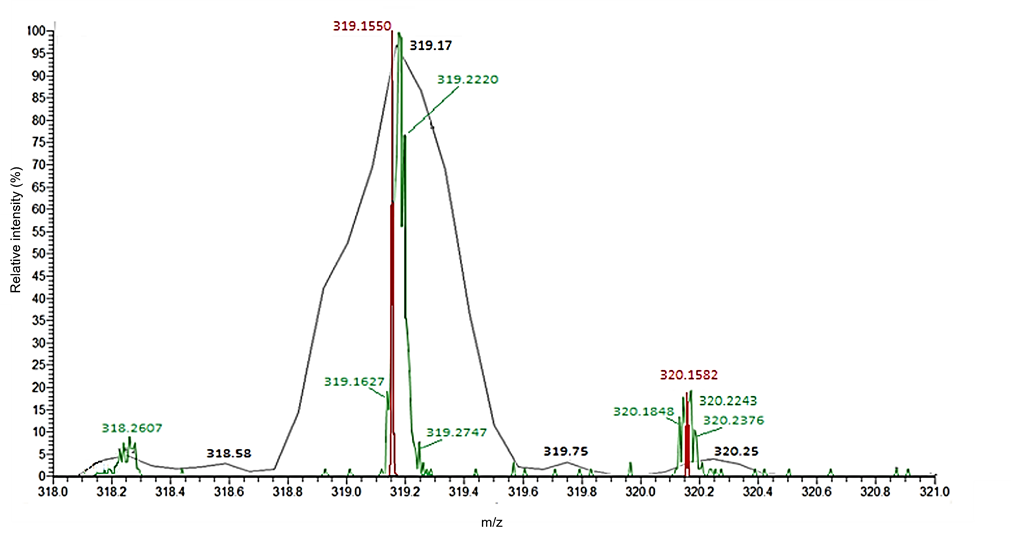


**Supplemental Figure S4.** Peak width differences for aZol parent ion (m/z = 319.1546) between LTQXL (black), the G1 in V mode (green), and the Orbi (red) using a 10 ng/mL standard spiked into urine


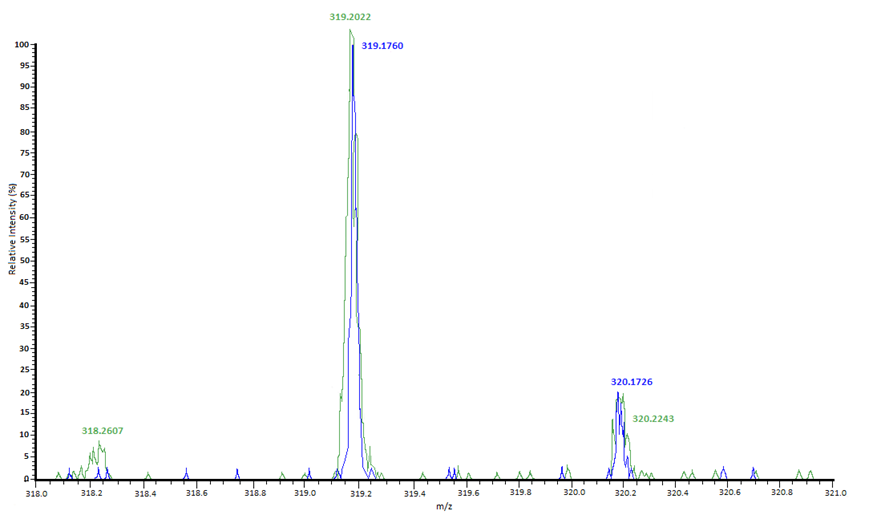


**Supplemental Figure S5**. aZol parent ion peak as observed on the G1 in V mode (green) and the G1 in W mode (blue showing minimal difference in peak width between the 2 modes. W mode has a manufacturer reported resolution ~ 2x > V mode

**Supplemental Table S1.** Optimized mass spectral conditions for each of the 4 platforms

|  | **LTQ** | **LTQXL** | **Orbi** | **G1 (V and W modes)** |
| --- | --- | --- | --- | --- |
| *Ionization source* | APCI negative | APCI negative | ESI negative | ESI negative |
| *Operation Mode* | SRM | SRM | PRM | MS/MS |
| *Vaporizer Temp (°C)* | 330.00 | 350.00 | - | - |
| *Sheath Gas Flow Rate* | 20 | 20 | 45 | - |
| *Aux Gas Flow Rate* | 5 | 5 | 15 | - |
| *Sweep Gas Flow Rate* | 5 | 5 | 0 | - |
| *Discharge Current (uA)* | 20.00 | 20.00 | - | - |
| *Spray Voltage (kV)* | - | - | 3.50 | 3.50 |
| *Capillary Temp (°C)* | 250.00 | 250.00 | 320.00 | - |
| *S-lens RF level* | - | - | 400.00 | - |
| *Aux Gas Heater Temp (°C)* | - | - |  | - |
| *Collision Gas* | Helium | Helium | Nitrogen | Argon |
| *Collision Energy* | 30 | 30 | 35 | 27.0 |
| *Capillary Voltage* | -44.00 | -6.00 | - | - |
| *Tube Lens (V)* | -103.22 | -78.20 | - | - |
| *Multipole 00 Offset (V)* | 1.00 | 0.00 | - | - |
| *Lens 0 Voltage (V)* | 0.00 | 0.00 | - | - |
| *Multipole 0 Offset (V)* | 3.25 | 2.50 | - | - |
| *Lens 1 Voltage (V)* | 30.00 | 6.00 | - | - |
| *Gate Lens Voltage (V)* | 78.00 | 62.00 | - | - |
| *Multipole 1 Offset (V)* | 18.00 | 16.00 | - | - |
| *Multipole RF Amplitude (V)* | 400.00 | 400.00 | - | - |
| *Front Lens (V)* | 3.50 | 3.00 | - | - |
| *Sampling Cone* | - | - | - | 40 |
| *Extraction Cone* | - | - | - | 6.0 |
| *Source Termperature (°C)* | - | - | - | 150 |
| *Desolvation Temperature (°C)* | - | - | - | 300 |
| *Cone Gas Flow (L/h)* | - | - | - | 20 |
| *Desolvation Gas Flow (L/h)* | - | - | - | 500 |
| *Source Gas Flow (mL/min)* | - | - | - | 15 |
| *Trap Gas Low (mL/min)* | - | - | - | 5.0 |
| *TOF Detector (V)* | - | - | - | 1990 |

**Supplemental Table S2.** Parent and product ions indicated in the MS method for all zeranol compounds on the LRMS (LTQ and LTQXL) and HRMS (Orbi and G1) platforms

|  | **LRMS** | | **HRMS** | |
| --- | --- | --- | --- | --- |
|  | **Parent Ion [M-1]** | **Product Ions** | **Parent Ion [M-1]** | **Product Ions** |
| *Zen* | 317.00 | 273.50, 299.50 | 317.1395 | 273.1497, 299.1288 |
| *Zer* | 321.00 | 277.50, 303.50 | 321.1708 | 277.1809, 303.1602 |
| *bZal* | 321.00 | 277.50, 303.50 | 321.1708 | 277.1809, 303.1602 |
| *aZol* | 319.00 | 275.50, 301.50 | 319.1552 | 275.1654, 301.1446 |
| *bZol* | 319.00 | 275.50, 301.50 | 319.1552 | 275.1654, 301.1446 |
| *Zan* | 319.00 | 275.50, 301.50 | 319.1552 | 205.0866, 275.1654, 301.1446 |
| *Zen-d_6_* | 323.00 | 279.50, 304.50 | 323.1773 | 279.1874, 304.1604 |
| *aZol-d_7_* | 326.00 | 282.50, 308.50 | 326.1992 | 282.2092, 308.1885 |

**Supplemental Table S3.** Extraction recovery (R_E_) calculations for SPE sample preparation. Calculations performed using a matrix-matched standard at 10 ng/mL. Average R_E_ at a lower, biologically relevant concentration of 0.025 ng/mL have also been provided to show consistent results independent of the concentration.

|  |  |  | **10 ng/mL** |  |  | **0.025 ng/mL (n=9)** |
| --- | --- | --- | --- | --- | --- | --- |
|  | **Experiment 1** | **Experiment 2** | **Experiment 3** | **Experiment 4** | **Average** | **Average (%CV)** |
| *Zen* | 117%  115%  78.3% | 80.57%  63.48%  65.49% | 86.51%  101.27% | 82.79%  83.26% | 87.4% | 89.5% ± 10% (**11.0%**) |
| *Zer* | 97.0%  91.6%  84.3% | 105.68%  89.50%  91.92% | 96.43%  118.28% | 78.36%  80.66% | 93.4% | 83.4% ± 15% (**17.7%**) |
| *bZal* | 94.6%  89.7%  83.0% | 79.95%  73.61%  72.42% | 112.91%  82.56% | 60.56%  62.42% | 81.2% | 93.3% ± 9% (**9.32%**) |
| *aZol* | 103%  99.2%  84.3% | 92.96%  73.41%  96.52% | 96.73%  114.94% | 81.67%  74.71% | 91.7% | 92.8% ± 9% (**10.1%**) |
| *bZol* | 105%  109%  84.2% | 76.95%  76.76%  72.56% | 105.26%  76.56% | 66.06%  68.06% | 84.0% | 92.4% ± 10% (**11.2%**) |
| *Zan* | 109%  103%  85.4% | 70.63%  64.89%  71.81% | 78.93%  89.59% | 81.69%  87.65% | 84.3% | 81.9% ± 23% (**28.3%**) |

**Supplemental Table S4.** Validation of the method used on the Orbi for real-world urine analysis. Additional calibration standards between 0-1 ng/mL were included to account for the anticipated low biological concentrations seen in urine. Experiments were conducted over the course of several days.

|  | **Linearity**  **(r)** | **LOD (ng/mL)** | **LOQ**  **(ng/mL)** | **Repeatability**  **(0.025 ng/mL; %CV)** | **Reproducibility (0.025 ng/mL; %CV)** |
| --- | --- | --- | --- | --- | --- |
| *n* | 9 | 9 | 9 | 10 | 7 |
| *bZal* | 0.980 | 0.033 | 0.111 | 17.2 | 20.6 |
| *bZol* | 0.987 | 0.019 | 0.062 | 6.50 | 22.1 |
| *Zer* | 0.991 | 0.005 | 0.018 | 13.2 | 12.3 |
| *aZol* | 0.988 | 0.067 | 0.225 | 18.8 | 13.8 |
| *Zan* | 0.999 | 0.024 | 0.079 | 2.10 | 15.7 |
| *Zen* | 0.999 | 0.084 | 0.279 | 4.00 | 7.35 |

**Supplemental Table S5.** A more detailed listing of instrument selections, method parameters, results, and overall summaries for previous literature utilizing triple quadrupole LC-MS platforms for zeranols analysis discussed in the text as **Table 3**.

| **Paper Title** | Confirmatory Analysis Method for Zeranol, its Metabolites and Related Mycotoxins in Urine by Liquid Chromatography-Negative Ion Electrospray Tandem Mass Spectrometry | Detection of Zearalenone and Its Metabolites in Naturally Contaminated Follicular Fluids by Using LC/MS/MS and in vitro effects of Zearalenone on Oocyte Maturation in Cattle | Detection of Six Zeranol Residues in Animal Derived Food by HPLC-MS/MS | Determination of zeranol, taleranol, zearalanone, a-zearalenol, b-zearalenol, and zearalenone in urine by LC-MS/MS | Biomonitoring of Mycotoxins in Urine: Pilot Study in Mill Workers | Simultaneous Determination of Ractopamine, Chloramphenicol, and Zeranols in Animal-Originated Foods by LC-MS/MS Analysis with Immunoaffinity Clean-Up Column | Exposure Assessment of Portuguese Population to Multiple Mycotoxins: The Human Biomonitoring Approach | Determination of Trace Zearalenone and Its Metabolites in Human Serum by a High-Throughput UPLC-MS/MS Analysis |
| --- | --- | --- | --- | --- | --- | --- | --- | --- |
| **Author (s)** | E.O. van Bennekom, L. Brouwer, E.H.M Laurant, H. Hooijerink, M.W.F. Nielen | M. Takagi, S. Mukai, T. Kuriyagawa, K. Takagaki, S. Uno, E. Kokushi, T. Otoi A. Budiyanto, K. Shirasuna, A. Miyamoto, O. Kawamura, K. Okamoto, E. Deguchi | T. Ding, J. Xu, F. Liu, C. Yang | I. Matraszek-Zuchowska, B. Wozniak, J. Zmudzki | W. Follmann, N. Ali, M. Blaszkewicz, G.H. Degen | X. Sun, Q. Tang, X. Du, C. Xi, B. Tang, G. Wang, H. Zhao | C. Martins, A. Vidal, M. De Boevre, S. De Saeger, C. Nunes, D. Torres, A. Goios, C. Lopes, R. Assuncao, P. Alvito | D. Sun, C. Li, S. Zhou, Y. Zhao, Y. Gong, Z. Gong, Y. Wu |
| **Year** | 2002 | 2008 | 2009 | 2013 | 2016 | 2017 | 2019 | 2019 |
| **Objective/Goals** | 1. Developing and validating an LC ESI MS method for determining all 6 zeranol forms following EU guidelines 2. Quantitation and qualitation | 1, measure concentrations of zeranol and its metabolites using LC-APCI-MS/MS 2. examine in vitro effects of Zeranol exposure in cattle | To develop an effective LC-MS/MS method to detect six zeranol residues | Develop a sensitive LCMS method for identification and quantification of related zeranol compounds | Biomonitoring study; to detect mycotoxin levels in a cohort of mill workers and compare it to controls to understand occupational exposure | Determine the co-occurrence of different mycotoxins in the same sample | 1. Assess exposure of the Portuguese population for 37 mycotoxins using urinary biomarkers 2. Characterize associated risk through exposure 3. analyze differences of urinary mycotoxin concentrations between urine samples of the same participant | To develop a high throughput, sensitive method for the quantitation of zeranol and its biomarkers in serum. |
| **Zeranol Compounds Analyzed** | Zan, Zer, bZal, Zen, aZol, bZol | Zen, aZol, bZol | Zan, Zer, bZal, Zen, aZol, bZol | Zan, Zer, bZal, Zen, aZol, bZol | Zen, aZol, bZol | Zan, Zer, bZal, Zen, aZol, bZol | Zan, Zer, bZal, Zen, aZol, bZol, aZol-GlcA, bZol-GlcA, Zen-14-GlcA, Zen-14-Sulf | Zan, Zer, bZal, Zen, aZol, bZol |
| **Internal Standards (IS)** | Zer-d4, bZal-d4 | - | - | Zer-d4, bZal-d4 | C^13^-Zen | - | C^13^-Zen | C^13^-Zen |
| **Matrix Analyzed** | bovine urine | bovine follicular fluids | animal derived foods (i.e. pork, eggs, milk) | animal urine | human urine | homogenized pork, fish, milk, liver | human urine | human serum |
| **Extraction Type** | C18 SPE | Strata C-18-E, Phenomenex SPE + IAC cleanups | MCX SPE | C18 SPE | ZearalaTest Immunoaffinity column | Immunoaffinity column (IAC-CRZ) | QuEChERS based procedure | Oasis PRiMEHLB µElutionplate |
| **LC Instrumentation** | Waters Alliance 2690 | 1200 series Agilent HPLC | Thermo Scientific Surveyor HPLC | Agilent 1200 series HPLC | Prostar Varian HPLC | Shimadzu LC-20 HPLC | Waters Acquity UPLC | Waters Acquity UPLC I-class |
| **Column** | C18 Waters Symmetry; 150x3mm, 5m | Waters SunFire C18; 150x2.1 mm, 5µm | Hypersil Gold; 150 x 2.1mm, 5µm | Inertsil ODS-3; 150x2.1mm, 3.1µm | Nucleosil 100-5 C18 HD; 125x3 mm | Shimadzu Shim-pack VP-ODS; 150x2.0mm, 5µm | Waters HSS T-3; 100x2.1mm, 1.8µm | CORTEC C18 Column; 2.1×100 mm, 1.6 µm |
| **Mobile Phase A** | H2O:ACN (90:10) | 70:30 ACN:H2O + 0.5mM ammonium acetate | H2O + 0.1% formic acid | MeOH:H2O (70:30) | 30% H2O | ACN | H2O: MeOH: Acetic Acid 94:5:1 + 5mM ammonium acetate | H2O |
| **Mobile Phase B** | H2O:ACN (10:90) | - | ACN | - | 70% MeOH | 2 mmol/L Ammonium Acetate + 0.2% formic acid | H2O: MeOH: Acetic Acid 97:2:1 + 5mM ammonium acetate | ACN:MeOH 20:80 |
| **Method Length (minutes)** | 26 | - | 14 | 20 | 18 | 15 (positive mode); 8 (negative mode) | 18 | 8 |
| **Column Temp (°C)** | 30 | 40 | - | 30 | 25 | 40 | - | 40 |
| **Flow Rate** | 0.4 mL/min | 1 mL/min | 250 µL/min | 200 µL/min | 0.3 mL/min | 0.3 mL/min | 0.3 mL/min | 0.4 mL/min |
| **Injection Volume (µL)** | 50 | 50 | 10 | 25 | - | 20 (positive mode); 10 (negative mode | - | 10 |
| **MS instrumentation** | Micromass Quattro Ultima tandem MS | API 2000 | Thermo Scientific TSQ Quantum Triple stage Quadrupole | Linear Ion Trap Quadrupole QTRAP5500 | Varian 1200-L Quadrupole | API4000 MS | Waters Quattro XEVO MS | Waters Xevo TQ-S tandem quadrupole MS |
| **instrument description** | Triple Quad | Triple Quad | Triple Quad | Triple Quad | Triple Quad | Triple Quad | HRMS | HRMS |
| **acquisition mode** | MRM acquisition | MRM acquisition | SRM | MRM acquisition | MRM acquisition | MRM acquisition | MRM acquisition | MRM acquisition |
| **ionization type** | negative ESI | APCI | negative ESI | negative ESI | negative ESI | positive and negative ESI | positive and negative ESI | negative ESI |
| **collision energy** | 22.0 eV | 36-51 V | 20-26 V | 28-46 eV | 16-26 eV | 17-35 V | 16-50 V | 20-32 eV |
| **LOD** | Zan(0.12) Zer (0.18), bZal (0.3), Zen (0.03), aZol (0.03), bZol (0.02) *units of ng/mL* | Zen (0.01), aZol (0.02), bZol (0.02) *units of µg/L* | all compounds 0.1 *units of µg/kg* | Zan(0.18) Zer (0.07), bZal (0.16), Zen (0.31), aZol (0.19), bZol (0.22) *units of µg/mL* | Zen(2), aZol(10), bZol (10) *units of ng/L* | Zan(0.02) Zer (0.03), bZal (0.03), Zen (0.03), aZol (0.04), bZol (0.04) *units of µg/kg* | Zan(0.15), Zer(1.12), bZal(1.6), Zen(0.2), aZol(0.61), bZol(0.91), aZol-GlcA(1.40), bZol-GlcA(1.72), Zen-14-GlcA(0.3), Zen-14-Sulf(2.6) *units of µg/L* | Zan(0.03), Zer(0.04), bZal(0.02), Zen (0.02), aZol (0.04), bZol (0.06) *units of ng/mL* |
| **LOQ** | - | - | all compounds 1.0 *units of µg/kg* | - | Zen(5), aZol(25), bZol (25) *units of ng/L* | Zan(0.05) Zer (0.09), bZal (0.09), Zen (0.09), aZol (0.10), bZol (0.10) *units of µg/kg* | Zan(0.32), Zer(3.8), bZal(4.2), Zen(0.8), aZol(1.4), bZol(2.1), aZol-GlcA(3.0), bZol-GlcA(3.5), Zen-14-GlcA(1.3) Zen-14-Sulf(4.9) *units of µg/L* | Zan(0.1), Zer(0.2), bZal(0.1), Zen (0.1), aZol (0.2), bZol(0.2) *units of ng/mL* |
| **repeatability (%CV or %RSD)** | Zan(19) Zer (17), bZal (7.3), Zen (16), aZol (27), bZol (10) | - | - | Zan(25.2) Zer (7.8), bZal (13.4), Zen (22.8), aZol (19.6), bZol (22.4) | - | - | - | - |
| **Accuracy (%)** | Zan(91.3) Zer (91.5), bZal (99.4), Zen (107.4), aZol (107.1), bZol (80.6) | - | - | - | - | - | - | - |
| **precision (%RSD)** | - | - | - | - | - | 1.2-9.1 | 7.5-15.8 | 2.53-8.22 |
| **Method detection rate (%)** | - | Zen alone or in combination with metabolites (25.0) | - | - | Zen (c:100, m:100, w:100), aZol (c:46, m:33, w:0), bZol (c:23, m:17, w:20) *c= control, m= men, w= women* | - | - |  |
| **Method recovery (%)** | - | - | - | Zan(84.4) Zer (100.0), bZal (81.3), Zen (71.7), aZol (85.3), bZol (96.8) |  |  | 89.2-109.6 (labeled as apparent recovery) | Method recovery 91.6-123.6% |
| **Extraction recovery (%)** | Zer-d4(51), bZAL-d4 (53) | Zen (108.5), aZol (95.5), bZol (103.6) | 65-115 | - | - | all compounds 75.6-101.1 | - | Zan(100.4), Zer(106.5), bZal(116.1), Zen(94.06), aZol(99.95), bZol(111.8) |
| **Summary** | Using 6 different animal urine samples, they determined that this LCMS method was capable of detecting and quantifying each of the compounds of interest. This was beneficial because most existing methods at the time were GC compatible, which had the downside of derivatization. They showed that the method and instrumentation was sensitive enough for the concentration range of interest, and predicted that it could be easily applied to zeranols in different matrices. | The three zeranol compounds could be detected from bovine samples using this LC/MS/MS method. Concentrations determined, though found in very low concentrations, and are dependent on the feeding habits on the farm. | Using animal products (i.e. eggs, milk, muscle) they were able to create an accurate, reproducible and sensitive LC-MS/MS method capable of separation and quantification of all 6 zeranol compounds of interest. | The method successfully identified and quantified the 6 related zeranol compounds, following the EU Commission requirements. | Urinary biomarker levels reflect mainly dietary exposure to mycotoxins. Additional occupational exposure for mill workers seems to be negligible in this particular case. | The method successfully separated, identified, and quantified all of the target mycotoxin compounds in animal products. While the method was applied to a set of real life samples and concentrations of all compounds were not detected, the authors feel that through their validation studies that this method is unique and can successfully be applied to a variety of matrices. | The 94 participants included in the study were shown to be exposed to 6 different mycotoxins by analyzing urine biomarkers. While the exposure was above safety risk calculations, it should still be looked at carefully due to some uncertainties that may have affected the calculation. | The researchers successfully developed and validated a high throughput LC-MS/MS method for identification and quantification of zeranol and its metabolites. When applied to real world samples, it was found that hardly any serum contained zeranol or its metabolites probably due to their fast excretion from blood. |

**Supplemental Table S6.** Limits of detection (ng/mL) of platforms using APCI ionization source.

|  | LTQ | LTQXL | **Orbi** |
| --- | --- | --- | --- |
| *Zen* | 0.202 | 0.154 | 0.0006 |
| *Zer* | 0.077 | 0.056 | 0.0230 |
| *bZal* | 0.028 | 0.025 | 0.0230 |
| *aZol* | 0.093 | 0.116 | 0.0023 |
| *bZol* | 0.242 | 0.99 | 0.0023 |
| *Zan* | 0.053 | 0.074 | 0.0060 |

**Supplemental Table S7.** Limits of detection (ng/mL) on the Orbi using both ionization sources. Results indicate that detection limits on the ESI are generally equivalent to 10x better than those determined using APCI.

|  | **ESI** | **APCI** |
| --- | --- | --- |
| *Zen* | 0.0006 | 0.0006 |
| *Zer* | 0.0023 | 0.0230 |
| *bZal* | 0.0230 | 0.0230 |
| *aZol* | 0.0006 | 0.0023 |
| *bZol* | 0.0006 | 0.0023 |
| *Zan* | 0.0023 | 0.0060 |

**Supplemental Table S8.** Variation (%CV) of zeranol peak area using both APCI and ESI ionization sources on the Orbi. All experiments were done on the same day using a matrix-matched urine standard at 0.025 ng/mL and 0.25 ng/mL (n = 3).

|  | **0.025 ng/mL** | | **0.25 ng/mL** | |
| --- | --- | --- | --- | --- |
|  | **ESI** | **APCI** | **ESI** | **APCI** |
| *Zen* | 3.59% | 5.67% | 16.8% | 16.7% |
| *Zer* | 11.6% | 16.2% | 15.9% | 1.93% |
| *bZal* | 0.46% | 25.1% | 15.6% | 11.5% |
| *aZol* | 0.99% | 10.6% | 20.9% | 0.60% |
| *bZol* | 3.84% | 7.67% | 18.3% | 5.58% |
| *Zan* | 7.63% | 16.9% | 15.2% | 1.75% |
